# Similar Structures to the E-to-H Helix Unit in the Globin-Like Fold are Found in Other Helical Folds

**DOI:** 10.3390/biom4010268

**Published:** 2014-02-27

**Authors:** Masanari Matsuoka, Aoi Fujita, Yosuke Kawai, Takeshi Kikuchi

**Affiliations:** 1 Department of Bioinformatics, College of Life Sciences, Ritsumeikan University, Kusatsu, Shiga 525-8577, Japan; E-Mails: cb006060@ed.ritsumei.ac.jp (M.M.); cb05066@is.ritsumei.ac.jp (A.F.); ykawai@sk.ritsumei.ac.jp (Y.K.); 2 Japan Society for the Promotion of Science (JSPS), Ichibancho, Chiyoda-ku, Tokyo 102-8471, Japan

**Keywords:** Globin-like fold, super-fold, keyword, dali search, average distance map

## Abstract

A protein in the globin-like fold contains six alpha-helices, A, B, E, F, G and H. Among them, the E-to-H helix unit (E, F, G and H helices) forms a compact structure. In this study, we searched similar structures to the E-to-H helix of leghomoglobin in the whole protein structure space using the Dali program. Several similar structures were found in other helical folds, such as KaiA/RbsU domain and Type III secretion system domain. These observations suggest that the E-to-H helix unit may be a common subunit in the whole protein 3D structure space. In addition, the common conserved hydrophobic residues were found among the similar structures to the E-to-H helix unit. Hydrophobic interactions between the conserved residues may stabilize the 3D structures of the unit. We also predicted the possible compact regions of the units using the average distance method.

## 1. Introduction

To elucidate how a protein folds into its unique 3D structure is a long-standing unsolved problem in molecular bioinformatics and molecular biophysics. To tackle this problem, we have to know how the information of a protein’s 3D structure construction is coded in its amino acid sequence. Protein folds are characterized by some combinations of secondary structural elements, and such structural characteristics of a protein are called its fold or topology. Some protein folds have neither sequence nor functional similarity. Proteins with these folds frequently appear in the structure database, that is, more than 30% of determined structures and such folds are called a “superfold” [1]. In such a case, one might ask if there is a common property in their amino acid sequences that leads to the same fold. Alternatively, is there a partial key 3D structure that leads to the same topology? If so, it should be a common 3D structural unit, which leads to a specific topology.

**Figure 1:**
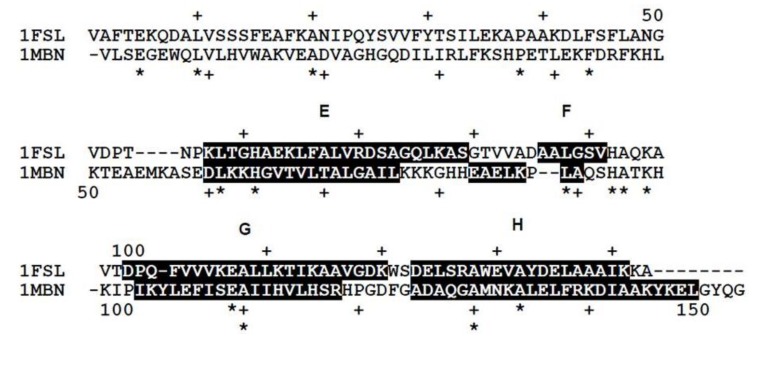
Amino acid sequence alignment of soybean leghemoglobin (PDB: 1FSL) and sperm whale myoglobin (PDB: 1MBN). The amino acid sequence identity is 15%. White letters with a black background denotes a residue in the α-helices of the E-to-H helix unit. The portions labeled by E, F, G and H refer to these α-helical regions.

**Figure 2:**
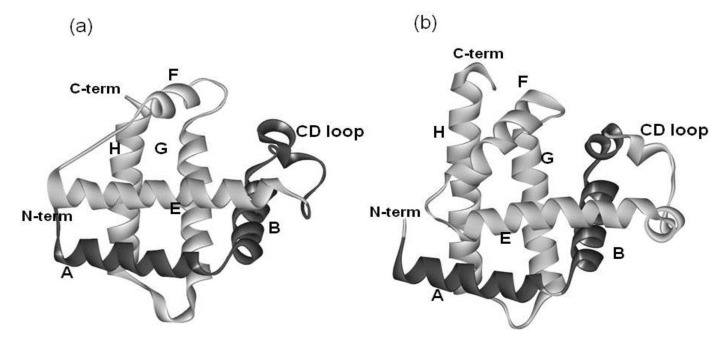
3D structures of (**a**) soybean leghemoglobin (PDB: 1FSL) and (**b**) sperm whale myoglobin (PDB: 1MBN). The portion in light gray is the E-to-H helix unit.

It is well-recognized that some of the proteins classified as having the globin-like fold according to the Structural Classification of Protein Database (SCOP) show a rather low amino acid sequence identity of around 15% in spite of the high conservativeness of the 3D scaffold [1]. The globin-like fold is regarded as a superfold [1]. Figure 1 and Figure 2 show the amino acid sequence alignment and 3D structures of leghemoglobin (soy bean) and myoglobin (sperm whale) as examples. The codes of the Protein Data Bank (PDB) are 1FSL and 1MBN, respectively.

**Figure 3:**
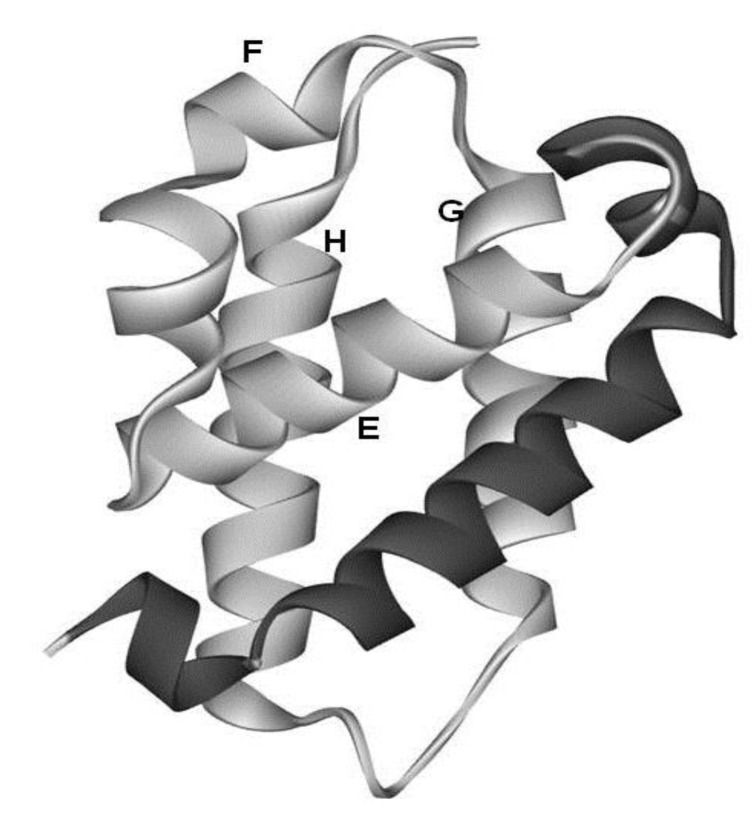
3D structure of 2-on-2 hemoglobin, i.e., *Mycobacterium tuberculosis* hemoglobin (PDB: 1NGK). The portion in light gray is the E-to-H helix unit. The E-to-H helix unit of this protein is very similar to that in 1FSL or 1MBN.

The amino acid sequence identity between them is 15%. As seen in Figure 2, a protein in the globin-like fold contains six helices A, B, E, F, G and H. Nakajima *et al.* [2] have revealed that the E-to-H helices is thought to be a folding initiation site, especially in the plant hemoglobins which are supposed to retain the ancestral characteristics in the predicted folding mechanism. This analysis corresponds well to the NMR experimental results [3] for leghemoglobin. Furthermore, a major part of the 3D structure of 2-on-2 hemoglobin (Mycobacterium tuberculosis), in which the N-terminal part in the peptide chain is truncated in comparison with leghemoglobin and myoglobin, is the E-to-H helix shown in Figure 3 [2].

These observations lead us to speculate that the E-to-H helix part forms an evolutionary conserved 3D scaffold, that is, this part is considered as a structure formation unit. In addition, proteins regarded as members of a superfold sometimes appear in several SCOP folds [1]. Thus, we have come to believe that the fold of the E-to-H helix unit may be widely observed in protein 3D structure space.

The purpose of the present study is to search for 3D structures similar to the E-to-H helix unit as independent folding units in SCOP folds besides the globin-like fold. Namely, we investigate the commonality of the 3D scaffold of the E-to-H helix unit in the protein 3D structure space. One technique employed in this work is a 3D structure alignment technique for the aforementioned purpose, the Dali algorithm [4]. Another technique employed involves a contact map constructed from only the amino acid sequence of a protein based on inter-residue average distance statistics (average distance map, ADM) [5,6]. This technique is used to predict a possible compact region in the 3D structure of a protein. In this study, a heme is not explicitly considered because a protein in the globin-like fold, myoglobin, has been confirmed to fold into its native structure without a heme [3].

A similar concept to the present study has been proposed as “protein lego” which refers to similar partial 3D structures observed in the protein 3D structure space [7,8]. Some analyses of 3D structural properties and stability of α-helix bundle were carried out by means of energy calculations [9,10,11,12,13,14,15,16,17,18,19], Wenxiang diagram and so on [20,21,22,23,24]. In the present work, we focus on the E-to-H helix unit predicting whether a corresponding part will form a compact region as a possible folding unit.

The organization of the present paper and the way of thinking are as follows. We first show the results of the DALI searches with the 3D structure of the E-to-H helix unit in soy bean leghemoglobin as a query followed by the ADM analyses for all hit proteins to examine whether the partial sequence of the E-to-H helix part in a hit protein exhibits a property to form a compact structure based on the ADM for this protein (Section 2.1). The details of the 3D structures of the proteins obtained in Section 2.1 are summarized in Section 2.2. In the Section 2.3, Section 2.4, and Section 2.5, the conservation of hydrophobic residues in homologues of a protein currently considered is presented and the hydrophobic packing formed by conserved hydrophobic residues is analyzed to examin whether a specific residue pattern can be observed in the E-to-H helix unit of a protein treated in the present study. Finally, the commonality of the E-to-H helix unit in the whole protein structure space is discussed.

## 2. Results

### 2.1. Dali Search and ADM Analyses

About 7500 hits were obtained by the Dali algorithm with the E-to-H helix unit of soybean leghemoglobin as a query. We picked up proteins with Z ≥ 2.0 so that the number of the false positive is less than about 5%. The hit proteins could be classified into the 690 SCOP families, and the representative protein from each family was selected based on the criterion described in the method section, Section 4.2. The 690 families are summarized in Table S1 of the Supplementary Material.

It is somewhat hard to pick up folding units by analyzing compact parts defined in 3D structures. Some proteins contain several compact parts, which may be probable folding units. In this study, in order to detect the property of partial peptide sequences, which tend to form compact structures, we use the ADM method. The ADM method predicts the possible compact regions in a protein from its amino acid sequence and predicted regions correspond well to structural domains [5,6,25], and experimentally observed folding regions including highly protected regions measured by NMR during folding [26,27,28].

**Figure 4:**
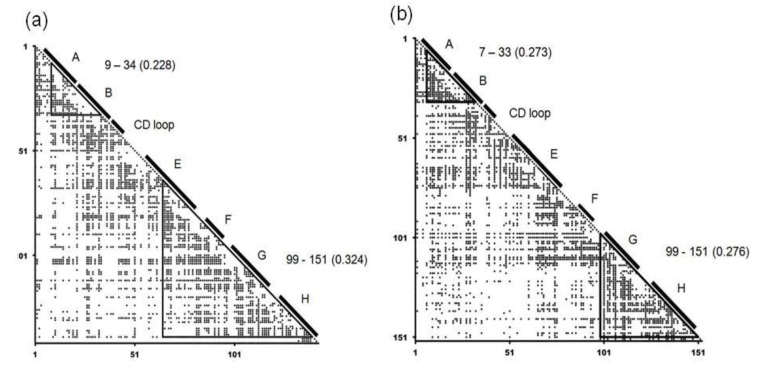
Average distance maps (ADM) for 1FSL (**a**), and 1MBN (**b**). The locations of the α-helices are labeled by A, B and so on. The label “9–34” denotes the compact region predicted by ADM. A numeral in parentheses indicates the η value of a compact region predicted by ADM.

For every representative protein, average distance map (ADM) analysis was performed and we took each protein in which the corresponding E-to-H unit was predicted as a compact region. An average distance map, ADM, is a kind of predicted contact maps and the details on ADM are described in the Section 4.2 (the method section). The ADMs for soybean leghemoglobin (PDB: 1FSL) and sperm whale myoglobin (PDB: 1MBN) are presented in Figure 4. In the ADM for soybean leghemoglobin, the regions 9–34 and 65–140 are predicted as compact regions with η values of 0.228 and 0.324 respectively. A η value denotes an index of the strength of the compactness of a predicted compact region by ADM (see method section for details). The respective predicted regions include helices A–B and E-to-H. From the η values, the E-to-H part can be regarded as the main compact unit in soybean leghemoglobin. On the other hand, the compact units 7–33 and 99–151 are predicted by the ADM for sperm whale myoglobin with the η values of 0.273 and 0.276 for these regions, respectively, with these regions corresponding to helices A–B and G–H as shown in Figure 4b. From the ADM for sperm whale myoglobin, the regions 7–33 and 99–151 are predicted to be equally compact. This prediction reflects the experimental results of folding of myoglobin by NMR [3,26]. This fact suggests that the G–H part sometimes strongly form in the E-to-H helix unit. Therefore, we also took each protein hit by the Dali search in which only the G–H part is predicted to be a compact region, because in sperm whale myoglobin only the G-H part is predicted to be a compact region.

The ADM predictions were applied for the representative proteins obtained above and finally, five representative proteins were obtained. We confirmed that these five proteins are also all obtained when the E-to-H helix unit from sperm whale myoglobin is used as a query instead of that from leghemoglobin (data not shown). The hit proteins and their details are summarized in Table 1.

**Figure 5:**
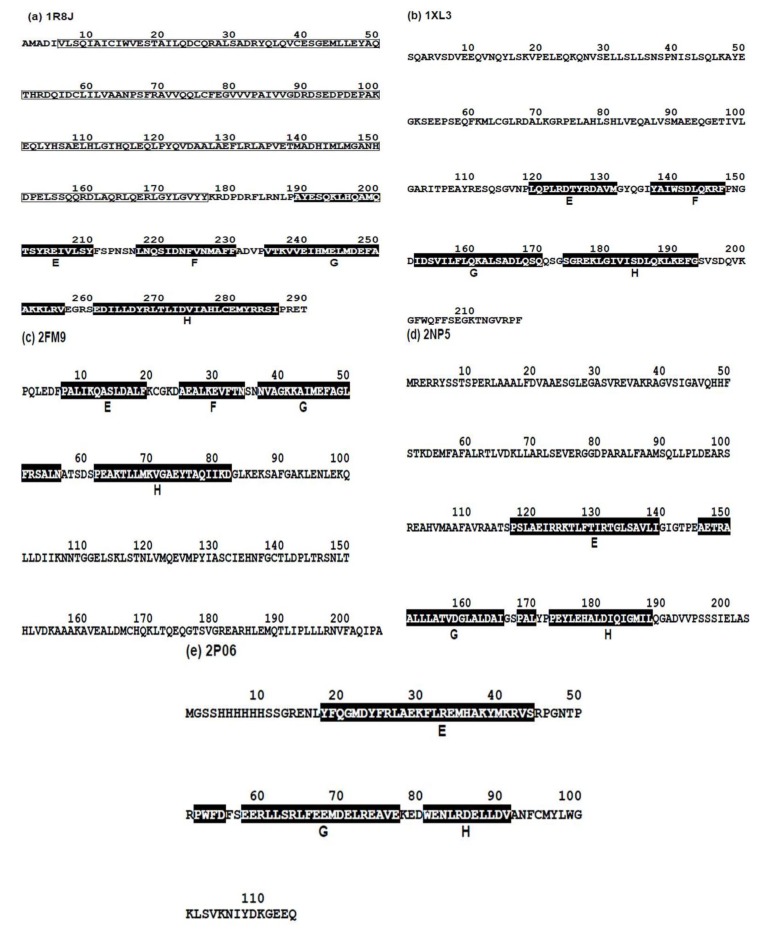
The amino acid sequences from FASTA files of (**a**) Circadian clock protein KaiA (Synechococcus, PDBID:1R8J), (**b**) Secretion control protein A chain (Yersinia, 1XL3), (**c**) Cell invasion protein SipA (Salmonella, PDBID: 2FM9), (**d**) Transcriptional regulator RHA1_ro04179 (Rodococcus, PDBID: 2NP5), and (**e**) Hypothetical protein AF0060 (*E. coli*, PDBID: 2P06). White letters with a black background denotes a residue in the α-helices of the corresponding E-to-H helix unit.

The amino acid sequences from FASTA files of these five proteins and the positions of the E-to-H helix parts are shown in Figure 5 (a helix is presented by white letters with a black background).

**Table 1:** Proteins obtained in the present work.

Protein(Source, PDB ID)	Family	Fold	Region Hit by DALI Search with the Following Query(Z Score, rmsd^(a)^)	
			Leghemoglobin E-to-H (Soy Bean, 1fsl)	**Myoglobin E-** **to-H (Sperm Whale, 1mbn)**
circadian clock protein Kai A (*Synechococcus*, 1R8J)	Circadian clock protein KaiA, C-terminal domain	KaiA/RbsU domain	E-to-H helices (4.4, 3.8)	E-to-H helices (7.0, 3.3)
secretion control protein A chain(*Yersinia*, 1XL3)	LcrE-like	Type III secretion system domain	EGH helices (2.0, 4.5)	FGH helices (4.1, 3.9)
cell invasion protein SipA (*Salmonella*, 2FM9)	SipA N-terminal domain-like	SipA N-terminal domain-like	E-to-H helices (2.9, 9.3)	H helix (3.0, 8.0)
transcriptional regulator RHA1_ro04179 (*Rodococcus*, 2NP5)	Tetracyclin repressor-like, C-terminal domain	Tetracyclin repressor-like, C-terminal domain	GH helices (4.4, 9.7)	GH helices (2.1, 4.7)
hypothetical protein AF0060 (*E. coli*, 2P06)	AF0060-like	all-alpha NTP pyrophosphatases	GH helices with a part of the E helix (3.2, 4.8)	GH helices (3.5, 3.5)

^(a): ^rms distance (Å) between the 3D structures of the aligned regions of the query structure and a hit protein.

### 2.2. Detailed Comparisons of the Folding Units Predicted by ADMs with a Region Hit by the Dali Search

The regions predicted by ADMs for the respective proteins with the η values are summarized in Table 2. The details are as follows. We may use a PDB ID to specify each protein.

**Table 2:** The summary of the results of the ADMs.

Protein (PDB ID)	Predicted Folding Unit	η Value
1FSL	9–34	0.228
	65–140	0.324
1MBN	7–33	0.273
	99–151	0.276
1R8J	5–34	0.218
	51–82	0.203
	223–270	0.254
1XL3	3–44	0.195
	77–99	0.202
	124–201	0.297
2FM9	1–51	0.292
	79–115	0.157
	166–199	0.297
2NP5	5–38	0.253
	76–103	0.175
	128–186	0.372
2P06	3–83	0.370

#### 2.2.1. Circadian Clock Protein KaiA (1R8J)

**Figure 6:**
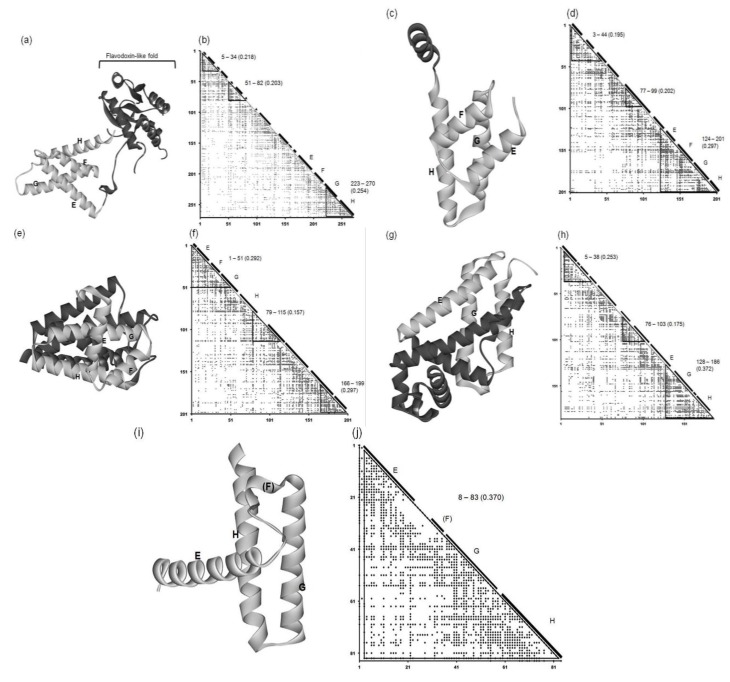
3D structures and ADMs of 1R8J (**a**) and (**b**), 1XL3(**c**) and (**d**), 2FM9 (**e**) and (**f**), 2NP5 (**g**) and (**h**), and 2P06 (**i**) and (**j**). A portion in light gray denotes the corresponding E-to-H helix part. The label “5–34” denotes the compact region predicted by ADM. The numeral in parentheses means the η value of a compact region predicted by ADM.

The 3D structure and ADM of the circadian clock protein KaiA (1R8J) are presented in Figure 6a and 6b. The predicted compact regions are 5–34,51–82 and 223–270 as shown in Figure 6b and the last predicted region with the highest *η* value corresponds to G and H helices in the E-to-H helix unit as presented in Table 2 and Figure 6b. The rest of this protein, that is, the region 1–179 contains a Flavodoxin-like fold domain, namely, α/β domain (see Figure 5a (regions enclosed by rectangles) and Figure 6a). Incidentally, the first and second predicted regions form the Rossmann fold.

#### 2.2.2. Secretion Control Protein (1XL3)

This protein consists of only α helices. Figure 6c and 6d present the 3D structure and ADM of the secretion control protein (1XL3). The predicted compact regions are 3–44, 77–99 and 124–201 and the corresponding E-to-H helices are included in the last predicted region with the highest η value (see Figure 6d and Table 2). The DALI search picked up the corresponding E, G and H helix parts in this protein, as the parts are structurally similar to the query structure. However, there is a helix between the corresponding E and G helices in this protein, and thus we regard this helix as the helix corresponding to the F helix (Figure 6d).

#### 2.2.3. Cell Invasion Protein SipA (2FM9)

This protein consists of only α helices. Figure 6e and Figure 6 f show the 3D structure and ADM of the cell invasion protein SipA (2FM9). The predicted compact regions are 1–51, 79–115 and 166–199 as presented in Table 2 and the region 1–51 corresponds to the E-to-H unit with one of the highest η values (0.292 for 1–51 and 0.297 for 166–199). The Dali search hit the segment corresponding to the E-to-G helices as the part structurally similar to the query structure (see also Figure S1 in Supplementary Material). The helix corresponding to the H helix is outside the predicted compact region, but the proximity of this helix to the E-to-G helix unit can be confirmed by visual inspection as seen in Figure 6e.

#### 2.2.4. Transcriptional Regulator RHA1_ro04179 (2NP5)

This protein consists of only α helices. The 3D structure and ADM of the transcriptional regulator RHA1_ro04179 (2NP5) are presented in Figure 6g,h. The predicted compact regions are 5–38, 76–103, and 128–186 as shown in Table 2 with the last predicted regions corresponding to the E (short part), G and H helices with the highest η value. The Dali search hit only the part corresponding to the G-H helices as the part structurally similar to the query structure (Figure S1 in the Supplementary Material). The other helix is perpendicularly close to these two helices, and this can be regarded as the E helix (see Figure 6g). There is no helix corresponding to the F helix.

#### 2.2.5. Hypothetical Protein AF0060 (2P06)

The 3D structure and ADM of the hypothetical protein AF0060 (2P06) are presented in Figure 6i and 6j. The ADM predicts the almost whole region to be the compact region (3–83) as seen in Table 2. The Dali search hit portions corresponding to the G-H helices and a small portion of the corresponding E helix as the part with a 3D structure similar to the query structure (Figure S1 in Supplementary Material). The structural similarity of this part to the E-to-H unit in leghemoglobin can also be confirmed by visual inspection (Figure 6i).

**Figure 7:**
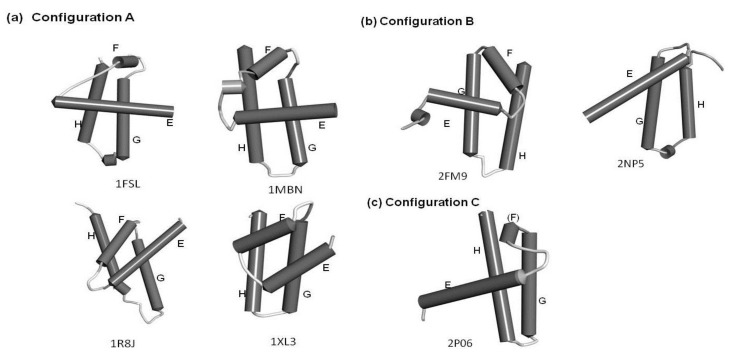
The configurations of helices E, F, G and H. (**a**) Configuration A. 1FSL, 1MBN, 1R8J and 1XL3 belong to this category. (**b**) Configuration B. Mirror image of configuration A. 2FM9 and 2NP5 belong to this category. (**c**) Configuration C. Variant of Configuration A. 2P06 belongs to this category.

The configurations of the four helices corresponding to the E-to-H helix unit in the proteins obtained by the present method are illustrated in Figure 7. In particular, 1R8J and 1XL3 have the same helix configuration as the helices in 1FSL and 1MBN. However, the helix configuration in 2FM9 and 2NP5 is almost a mirror image of that in 1FSL. This is because the Dali search picks up a protein with a 3D structure similar to that of a query protein by comparing the inter-Cα distances, and thus a protein with a mirror image structure with a query can be hit. 2P06 contains a helix configuration similar to that of 1FSL, but the orientation of the helix E is different. Thus, one should be careful about the helix configuration in each E-to-H helix unit. We call these helix configurations Configuration A (the configuration in 1FSL, 1MBN, 1R8J, and 1XL3), Configuration B (the configuration in 2FM9 and 2NP5), and Configuration C (configuration in 2P06), respectively, as illustrated in Figure 7.

### 2.3. Conserved Residues in the E-to-H Unit

We performed BLAST searches for amino acid sequences of the E-to-H helix parts in five proteins with leghemoglobin and myoglobin followed by multiple alignments with MUSCLE (see method section, Section 4.4, for details). The conservation in a helix corresponding to the F helix in 1FSL or 1MBN was not taken into account because this helix is not indispensable for structure formations [3]. The statistics of the searched homologs are summarized in Table 3. The accession codes of homologues and their multiple aliments are presented in Figure S2 of the Supplementary Material. Because there is no sufficient amino acid sequence diversity among the homologous sequences of 2FM9 and 2NP5, we cannot find any residues, which could be identified as the conserved residue in terms of the number of amino acid substitutions. The conservation in the amino acid sequence of 2P06 could not be evaluated since there is no significant hit with the amino acid sequence of this protein as a query on UniprotKB databases by a BLAST search. The configurations of the helices in these proteins, 2FM9, 2NP5, and 2P06, are shown in Figure 7b and 7c. Thus, we discuss the conservation of residues for the following four proteins: 1FSL, 2MBN, 1R8J, and 1XL3. All of these proteins contain the E-to-H helix unit with Configuration A (Figure 7a).

**Figure 8:**
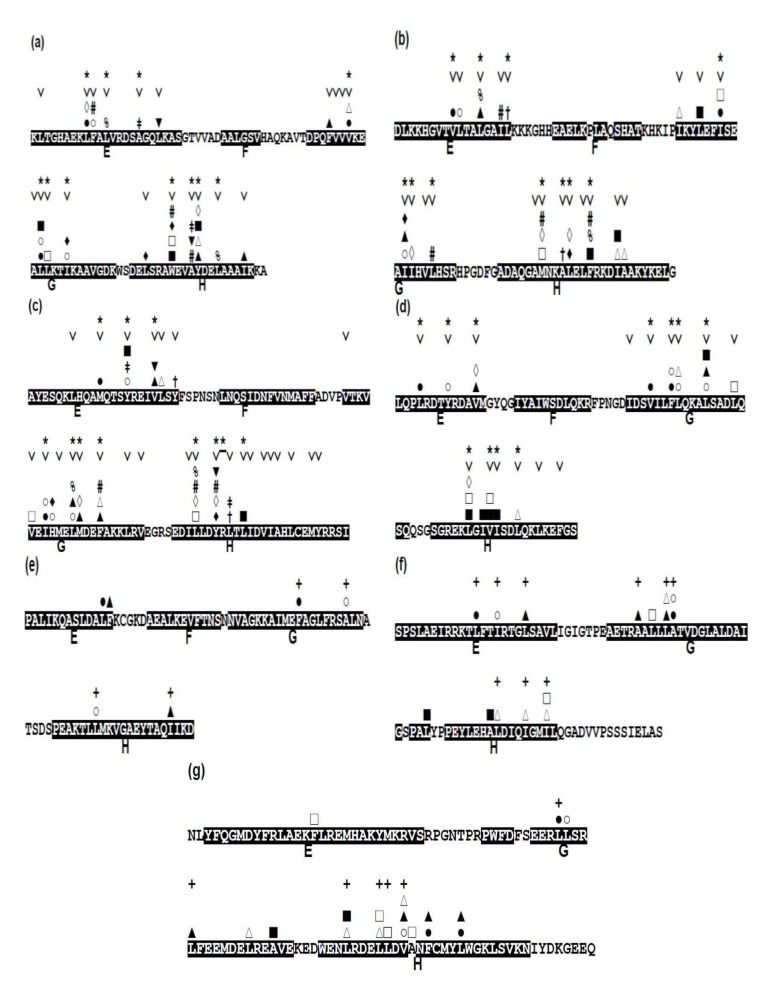
Conserved hydrophobic residues in the E-to-H helix unit of (**a**) 1FSL, (**b**) 1MBN, (**c**) 1R8J, (**d**) 1XL3, (**e**) 2FM9, (**f**) 2NP5, and (**g**) 2P06. The conserved residues are labeled with the mark “˅”. Any two residues with the same mark (one of the marks, #, %, ‡, †, ▲, ▼, ■, □, ○, ◊, and ∆) in different helices denote that this residue pair makes a hydrophobic packing detected by the buried surface. Residues with “*” in a helix constitute a common residue pattern specific for the E-to-H helix unit. The residue with “*” actually does not form hydrophobic packing. For (**e**) 2FM9, (**f**) 2NP5 and (**g**) 2P06, a residue with a mark “+” indicates a residue pattern similar to one of the present common residue patters in Table 4.

Focusing on the conservativeness of hydrophobic residues, that is, A, F, I, L, M, V, and W, we present the conserved hydrophobic residues in each protein in Figure 8, indicating the conserved residues with the mark “˅”.

**Table 3:** Homologues of the proteins in the present analyses.

Protein(Source, PDB)	Number of Homologs	Number of Conserved Residue	Number of Residues Containing E-to-H Helices
Leghemoglobin (soybean 1FSl)	45	34	88
Myoglobin (sperm whale 1MBN)	82	38	96
Circadian clock protein KaiA (Synechococcus, 1R8J)	49	50	98
Secretion control protein) A chain (Yersinia, 1XL3)	29	25	76
Cell invasion protein SipA (Salmonella, 2FM9)	6	0	79
Transcriptional regulator RHA1_ro04179 (Rodococcus, 2NP5)	3	0	74
Hypothetical protein AF0060 (*E. coli*, 2P06)	0	0	81

### 2.4. Residues Involved in Hydrophobic Packing Assigned Based on Buried Surface

The packing residue pairs in the E-to-H unit in each protein detected based on buried surface (see method section, Section 4.3, for details) are depicted in Figure 8. In this figure, any two residues with the same mark (one of the marks, #, %, ‡, †, ▲, ▼, ■, □, ○, ◊, and ∆) in different helices denote that this residue pair makes hydrophobic packing. For the analyses of the packing residue pairs, the F helix was not considered in the present study because it has been confirmed that the F helix in leghemoglobin does not play a significant role in its folding as already mentioned [3]. Relatively many packing pairs are observed between the G and H helices in every protein while a few packing residues are observed between the E and G or H helices as seen in Figure 8.

From this figure, we can confirm that the almost all the packing hydrophobic residue pairs are distributed in the conserved residues. Only 1 of the 58 packing residues, 181-I in 1XL3, are not conserved residues in the present definition (Figure 8, see also Figure 5b).

### 2.5. Common Residue Patterns Specific to the E-to-H Helix Unit Defined from the Packing Patterns of Conserved Hydrophobic Residues

**Figure 9:**
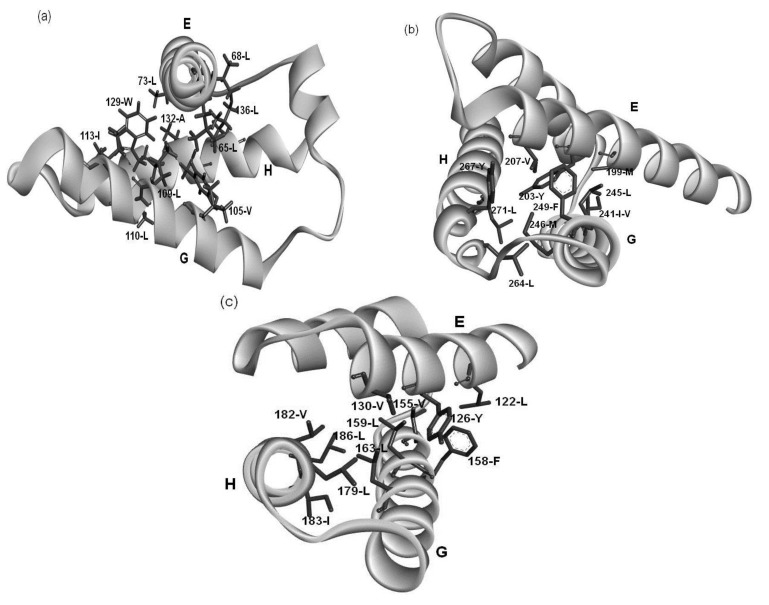
The hydrophobic packing formed by residues of the common patterns in (**a**) 1FSL; (**b**) 1R8J and (**c**) 1XL3.

A common residue pattern might be defined from the information of both conserved hydrophobic residues and the packing residues of the E-to-H helix unit in 1FSL, 1MBN, 1R8J and 1XL3. The common residue patterns defined by visual inspection are presented in Figure 8. A residue constituting a common pattern is labeled by the mark “*”. These residue patterns are also summarized in Table 4. The common residue patterns are expressed as φx(3)φx(3)φ [or φx(2)φx(4)φ] for the E helix, φx(2,3)φ(2)x(2, 3)φ for the G helix, φx(2)φ(2)x(2)φ for the H helix. The symbol φ denotes a hydrophobic residue. Only 1of the 44 residues in these patterns, 267-R in 1R8J (a residue with the mark “*” in Figure 8), does not form hydrophobic packing. The packing of the residues constituting common residue patterns in the E-to-H helix unit in 1FSL, 1R8J and 1XL3 is presented in Figure 9a,c respectively.

For 2FM9, 2NP5, and 2P06, the similar residue patterns are also inferred by visual inspection based on only the packing in Figure 8e,g. A residue in such a pattern in 2FM9, 2NP5, and 2P06 is labeled by “+” in Figure 8e,g. These residue patterns are also summarized in Table 4. The residue patterns for 2FM9, 2NP5, and 2P06 deviate somewhat from those in the four proteins discussed above. This is probably due to the different configurations of the E-to-H helix units [Figure 7b,c] compared with the helix configuration of the four proteins 1FSL, 1MBN, 1R8J, and 1XL3. Thus, the rule of common residue patterns cannot be applied strictly to 2FM9, 2NP5, and 2P06 as presented in Table 4.

**Table 4:** Common residue patterns.

Protein	E Helix	G Helix	H Helix
1FSL	φx(2)φx(4)φ	φx(3)φ(2)x(2)φ	φx(2)φ(2)x(2)φ
1MBN	φx(3)φx(3)φ	φx(3)φ(2)x(2)φ	φx(2)φ(2)x(2)φ
1R8J	φx(3)φx(3)φ	φx(3)φ(2)x(2)φ	φx(2)φx(3)φ
1XL3	φx(3)φx(3)φ	φx(2)φ(2)x(3)φ	φx(2)φ(2)x(3)φ
2FM9	—	φx(6)φ	φx(10)φ
2NP5	φx(2)φx(3)φ	φx(3)φ(2)x	φx(3)φ(2)x(2)φ
2P06	—	φx(3)φ	φx(3)φ(2)x(1)φ

The location of these residue patterns for 1R8J, 1XL3, 2FM9, 2NP5, and 2P06 correspond well to that for 1FSL. Figure S1 in Supplementary Material shows the amino acid sequence alignments based on the Dali algorithm of these proteins with 1FSL. In this figure, a residue with the mark “†” constitutes a common residue pattern.

## 3. Discussion

The results of the present study show that the common topology of the E-to-H helix unit can be observed beyond the globin-like fold when the Dali search is performed followed by ADM screening to check the tendency of a partial amino acid sequence of the hit regions to form a compact structure. This finding demonstrates that the E-to-H helix unit is a common structural unit in the structural space of proteins. Thus, E-to-H structures appearing in several protein folds must not necessarily have evolved from one common ancestor. Instead, the 3D structures of the E-to-H unit should be energetically stable and so this unit is observed widely in the 3D structure space. As far as the globin-like fold is concerned, as mentioned in the introduction section, there are truncated hemoglobins as primitive hemoglobins and the main structural unit of these proteins is the E-to-H helix unit part. It is speculated that E-to-H unit must be the basic structure in the early stage of the evolution of globin-like fold proteins, and this unit might grow to various globin-fold proteins during evolution. Proteins in other folds, for example, 1R8J, 1XL3 and so on, might also have grew from each ancestor protein with a structure similar to the E-to-H helix unit during evolution. Among the hit proteins, 1R8J is interesting because this protein is composed of two domains, one is the E–to-H unit and the other is the domain, while the other hit proteins are all proteins. This also indicates that the E-to-H helix unit widely exists in the structural space of proteins.

Examining the conservation of packing residues with respect to the homologues of each protein reveals the common patterns of packing residue locations on the helices. These residue patterns seem to be typical patterns in -helices, but the present specific combination of these residue patterns must stabilize the 3D structure of the E-to-H helix unit. In other words, we speculate that the helix bundle formed by the G and H helices is stabilized and the E helix interacts with this bundle as shown in Figure 9. It might be possible to define a structural motif specific for E-to-H helix unit by sophisticating the present amino acid sequence patterns.

For the other hit proteins with the Configurations B and C, only loosely defined common residue pattern are observed as seen in Table 4. Thus, proteins with Configurations B and C show the irregularity of the patterns. Proteins with the Configurations B and C may be excluded from hit proteins by Dalilite by tuning of a Z-score.

The analyses in the present study may be extended to other protein structural units and may lead us to find various common structural units beyond folds. We are currently studying along this direction.

## 4. Method

### 4.1. Search Protein 3D Structures Similar to that of the E-to-H Helix Unit in Leghemoglobin

In order to search for a 3D structure similar to that of the E-to-H helix unit, a 3D structure comparison program, Dalilite (http://ekhidna.biocenter.helsinki.fi/dali/star), was used [4] with the PDB structures. The 3D coordinates of the Cα atoms in the E-to-H units in soy bean leghemoglobin (PDB: 1FSL) were used as a query because the E-to-H unit in this protein has been confirmed as a folding core [2,3,26]. To do this, we prepared a 3D structure database, with Dalilite searches for the E-to-H-unit-like structures, by excluding the globin-like fold proteins from the whole PDB structures. Because all the globin-like fold proteins are expected to potentially possess an E-to-H unit, any PDB structures annotated as “globin-like fold” at SCOP were discarded from the Dali search. The protein in which the longest region aligned with the query structure produced by the Dalilite search in comparison with the other proteins within a SCOP family was taken as the representative of this family.

### 4.2. Prediction of Compact Regions Based on the Average Distance Map Method

To examine whether a polypeptide chain with the E-to-H-unit-like structure in a hit protein by Dalilite itself tends to form a compact structure, we employ the average distance map method. The summary of this method is presented as follows. The readers can also refer to [5,6] for more details.

For a given amino acid sequence with an unknown 3D protein structure, a kind of predicted contact maps is defined by plotting a possible contact on a map when the average distance of a pair of residues in a “range” is less than a cutoff value determined beforehand. A map defined with inter-residue average distance statistics is referred to as Average Distance Map (ADM) in this paper.

The procedure to construct ADM is presented as follows.

(1) Calculations of average distances.

The average distances between a pair of Cρ atoms of residues were calculated in each range using proteins with known structures. A range is defined by the length between two residues along the amino acid sequence of a given protein. The range *M* = 1 is defined when 1 ≦ *k*≦ 8, where *k* = |*i* − *j*| for *i*-th and *j*-th residues along the amino acid sequence. In the same way, the respective ranges *M* = 2, 3, 4 were defined by 9 ≦ *k* ≦ 20, 21 ≦ *k* ≦ 30, 31 ≦ *k* ≦40 and so on. For every range, the inter Cα atomic distances between two residues were calculated using proteins with known 3D structures [5].

(2) Construction of ADM.

To construct a map for a given amino acid sequence, a cutoff distance for each range should be defined. These values are defined so that the contact density of the whole real distance map (RDM) of the protein is reproduced [5]. The RDM refers to a typical contact map, in other words, a map constructed based on the determined 3D structure of a protein. In the present study, an RDM corresponds to a map constructed with a cutoff of inter-residue Cα atomic distance of 15 Å. The formula, 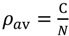, where *N* is the total number of residues of a protein and C is an adjustable constant, approximately predicts the average values of the contact density of the entire region of an RDM [5]. C = 36.12, which corresponds to the 15Å threshold for the construction of an RDM, is used in the present work [5]. The cutoff distances for the construction of an ADM from the amino acid sequence of a protein are determined to reproduce a value of 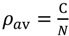. A different cutoff distance is found for a different range to construct an ADM, whereas in the case of an RDM construction just one cutoff distance is required. In the construction of an ADM, it is assumed that the number of residue pairs that make contacts (and should therefore be plots on a map) obeys the following Equation in a range *M* [5]:

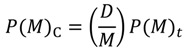


Here, *P*(*M*)_C_ is the number of amino acid pairs whose average distances in the range M is less than a given cutoff distance (i.e., residue pairs to be plotted on the ADM), and *P*(*M*)*_t_* is the total number of residue pairs in a given range (i.e., the number of the pairs with statistical significance among the whole 210 pairs of residues [5].

*D* is an adjustable parameter chosen to adjust the overall average density *ρ_av_* of the ADM to be around the predicted value of *ρ_av_* on an RDM. For an amino acid sequence of a protein, when a pair of residues has an average distance less than the cutoff distance in the range of the residue pair, a plot is made on a contact map. In this way, based on the inter-residue average distances, a kind of predicted contact map is produced from only the amino acid sequence of a given protein.

A constructed map is analyzed by the following procedure.

(1) Calculation of contact density differences.

**Figure 10:**
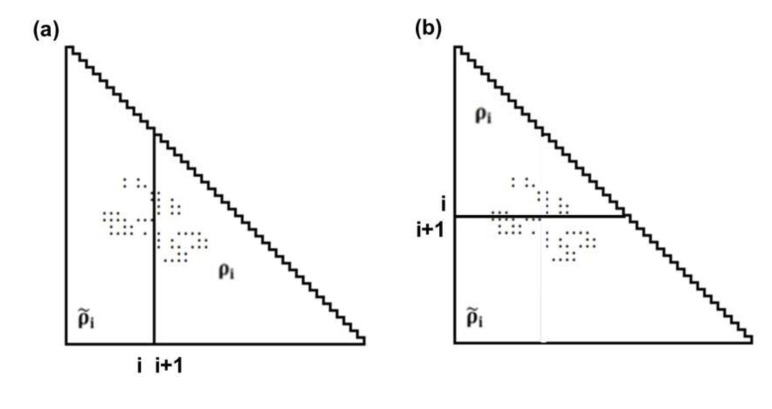
Schematic drawing of a map divided by a line parallel to the ordinate at the *i*-th residue (**a**), and divided by a line parallel to the abscissa at the *i*-th residue (**b**). The density of plots in the trapezoidal part and the triangular parts are denoted by 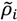 and *ρ_i_*, respectively.

Suppose that *ρ_i_* and 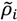 represent the contact density of the triangular and trapezoidal parts, respectively, when the whole area of a map is divided into two parts by a line parallel to the abscissa at the *i*-th residue or by a line parallel to the ordinate at the *i*-th residue as illustrated in Figure 10a and 10b. The contact density difference value is defined as 
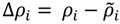
.

The values of the contact density difference, Δ*ρ_i_* are calculated from residues 1 to N, providing a scanning plot of Δ*ρ_i_*. The scanning plot produced by the division using the line parallel to the ordinate is called horizontal scanning, and the plot produced by the line parallel to the abscissa is called vertical scanning. *h* of Δ*ρ_i_^h^* and *v* of Δ*ρ_i_^v^* denote the horizontal and vertical divisions of a map, respectively.

(2) Detection of boundaries of a compact region.

**Figure 11:**
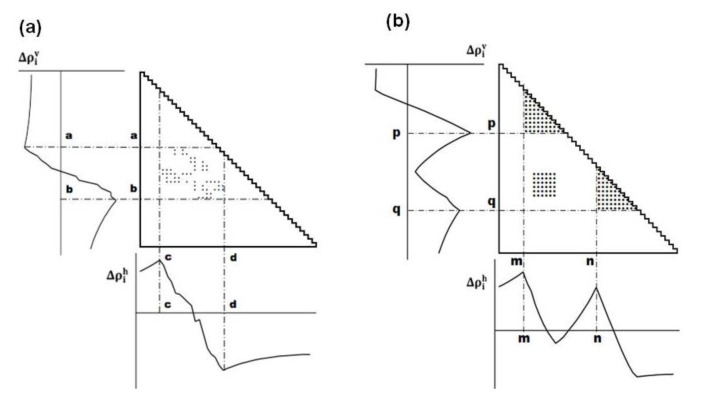
(**a**) Schematic drawing of a map with some plots. A peak and a valley appear at the boundaries of a highly dense region of plots. This map suggests the interaction between the segments *a*–*b* and *c*–*d*. (**b**) Hypothetical contact map with two compact areas near the diagonal along with the horizontal and vertical scanning plots. This map suggests the existence of two domains at the regions *p*–*q* and *m*–*n*. We define *η* as a measure of the compactness of the region, namely, 
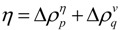
 or 
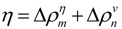
.

The maximum (peak) or minimum (valley) in the scanning plot reflects a large change in contact density values on a map. Figure 11a shows a schematic example of a horizontal scanning plot of Δ*ρ_i_^h^* from 1 to *N*, and at the bottom of the figure, a peak and a valley appear at *c* and *d*, respectively indicating a large change in contact density values. The same situation is observed in the vertical scanning in Figure 11a, indicating a peak and a valley at *a* and *b*, respectively (shown the left of the figure). The boundary of a compact region on a map can be detected by a peak and a valley appearing in the horizontal and vertical scanning plots of contact density differences.

(3) Prediction of location of compact regions.

The following explains how to define a possible compact region on a map using the positions of the scanning plot peaks. Figure 11b shows a hypothetical contact map with two compact areas near the diagonal with the horizontal and vertical scanning plots. We recognize the existence of two domains by the peaks at residues *m* and *n* in the horizontal scanning plot and residues *p* and *q* in the vertical scanning plot. Thus, the regions *m*–*p* and *n*–*q* on the map are predicted as possible compact regions or domains in the protein. In the ADM method, the strength of the compactness of a region *m*–*p* is measured by *η* values defined by 
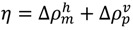
 (Figure 11b) [5].

The procedure described above predicts the regions of possible compact regions in a protein from only its amino acid sequence. The region with the highest *η* value can be defined a most probable compact region and such a region can be interpreted as a folding unit in a protein.

### 4.3. Identification of Residues Forming Hydrophobic Packing

The residues forming the hydrophobic packing were identified by the decrease of solvent accessible surface area (SASA) upon folding. If the decreases in SASA exceed 10 Å^2^ in both of a given two residues, this pair of residues is regarded as hydrophobic packing residues. The reduction in the surface area is given by the difference between the surface area of the side chain atoms in the presence of a contact with another residue upon folding and that in the absence of a contact with another residue before folding. The SASAs were numerically calculated by enumerating the grid points dropped onto the mesh surface equidistant from each atom of a side chain [29]. The distance of a grid point from the center of an atom is the van der Waals radius of each atom plus 1.4 Å, which is the probe radius corresponding to van der Waals radius of a solvent molecule (i.e., water). We confirmed that this criterion residue pair packing corresponds to the contact of two respective atoms in each residue within approximately 5 Å.

### 4.4. Identification of Conserved Residues in the E-to-H Helix Unit in Each Protein

In order to identify the conserved residues across the E-to-H helix unit of each protein hit by the Dali search, homologous amino acid sequences of the helix unit were collected from the amino acid sequence database, UniProt (http://www.uniprot.org/), by means of BLAST. The amino acid sequence of the E-to-H helix unit of a representative protein for each family as well as that of leghemoglobin (1FSL) were queried against the UniRef100 which is non-redundant set of the UniProt database. A threshold of the expected value of a BLAST search for a hit amino acid sequence retrieval was taken sufficiently low value, *e* = 0.001, to ensure that homologous sequences were obtained.

We made a multiple alignment of the obtained amino acid sequences for each query protein using MUSCLE [30]. Because, in general, the cause of the high similarity among homologous amino acid sequences cannot be distinguished either by the structural conservation or by a phylogenetic closeness, we conducted the phylogenetic analysis to find conserved residues across the homologous amino acid sequence caused by 3D structural restraints by comparing the number of amino acid substitutions in each residue. The phylogenetic trees of homologous amino acid sequences were reconstructed to obtain their ancestral amino acid sequences. The phylogenetic reconstruction was carried out by the NJ method implemented in Seaview [31]. The ancestral amino acid sequence estimation was carried out by PAML4.0 [32] where the reconstructed tree was used as the input tree and the JTT model was used as the amino acid substitution model. The number of amino acid substitutions through the tree was determined for every residue from the estimated ancestral amino acid sequences. Since the total length of the phylogenetic tree corresponds to the expected number of amino acid substitutions averaged over all residues and the distribution of the number of amino acid substitutions can be approximated using the Poisson distribution, the residue at a position in a multiple alignment, which shows that the number of amino acid substitutions at this position is within 5% from the lower level expected from Poisson distribution with mean values estimated from tree length, can be regarded as the genuine conserved residue.

## Supplementary Files

Supplementary File 1 Supplementary Information (PDF, 1525 KB)
