# Supplementary material for: Similar Structures to the E-to-H Helix Unit in the Globin-Like Fold are Found in Other Helical Folds
Source: Biomolecules. 2014 Feb 27;4(1):268–88. doi: 10.3390/biom4010268 (PMC4030988; doi:10.3390/biom4010268)

Table S1: All Families hit by the present Dali search

14-3-3 protein
1-deoxy-D-xylulose-5-phosphate reductoisomerase, C-terminal domain
2Fe-2S ferredoxin domains from multidomain proteins
3-Methyladenine DNA glycosylase III (MagIII)
5-carboxymethyl-2-hydroxymuconate isomerase (CHMI)
5' nucleotidase-like
5' to 3' exonuclease catalytic domain
5' to 3' exonuclease, C-terminal subdomain
6-phosphogluconate dE-Hydrogenase-like, N-terminal domain
ABC transporter ATPase domain-like
Aconitase B, N-terminal domain
Aconitase iron-sulfur domain
Actin/HSP70
Acyl-CoA binding protein
acyl-CoA oxidase C-terminal domains
acyl-CoA oxidase N-terminal domains
Adapter protein APS, dimerisation domain
Adenylation domain of NAD+-dependent DNA ligase
Adenylyl and guanylyl cyclase catalytic domain
Adenylylcyclase toxin (the edema factor)
a domain/subunit of cytochrome bc1 complex (Ubiquinol-cytochrome c reductase)
Aerobic respiration control sensor protein, ArcB
AF0060-like
AF0941-like
AF1104-like
AgoG-like
Alcohol dE-Hydrogenase-like, C-terminal domain
Alcohol dE-Hydrogenase-like, N-terminal domain
AldE-Hyde ferredoxin oxidoreductase, C-terminal domains
AldE-Hyde ferredoxin oxidoreductase, N-terminal domain

ALDH-like
Allosteric chorismate mutase
alpha-Amylases, C-terminal beta-sheet domain
alpha-catenin/vinculin
alpha-D-glucuronidasE-Hyaluronidase catalytic domain
alpha-D-glucuronidase, N-terminal domain
Alpha-hemoglobin stabilizing protein AHSP
alpha-Subunit of urease
alpha-subunit of urease, catalytic domain
Amidase signature (AS) enzymes
A middle domain of Talin 1
Aminoacid dE-Hydrogenase-like, C-terminal domain
Aminoacid dE-Hydrogenases
Aminoglycoside phosphotransferases
AmyC C-terminal domain-like
AmyC N-terminal domain-like
Amylase, catalytic domain
Animal lipoxigenases
Anticodon-binding domain of a subclass of class I aminoacyl-tRNA synthetases
Antioxidant defense protein AhpD
Anti-sigma factor AsiA
Apolipophorin-III
Apolipoprotein
Aquaporin-like
Archaeal DNA-binding protein
Arfaptin, Rac-binding fragment
Arginyl-tRNA synthetase (ArgRS), N-terminal 'additional' domain
Aristolochene/pentalenene synthase
Armadillo repeat
Arp2/3 complex 16 kDa subunit ARPC5
Arylsulfatase

Aspartate/ornithine carbamoyltransferase
Aspartokinase allosteric domain-like
ATP synthase
ATP synthase (F1-ATPase), gamma subunit
a tRNA synthase domain
Atu0492-like
Avirulence protein AvrPto
B12-dependent (class II) ribonucleotide reductase
Bacterial dinuclear zinc exopeptidases
Bacterial exopeptidase dimerisation domain
Bacterial GAP domain
Bacterial glucoamylase C-terminal domain-like
Bacterial glucoamylase N-terminal domain-like
Bacterial photosystem II reaction centre, L and M subunits
Bacterial tryptophan 2,3-dioxygenase
Bacteriophage CII protein
Bacteriorhodopsin-like
BAG domain
Band 7/SPFH domain
BAR domain
Barrier-to-autointegration factor, BAF
BAS1536-like
BC ATP-binding domain-like
BC C-terminal domain-like
Bcl-2 inhibitors of programmed cell death
BC N-terminal domain-like
Beta-D-glucan exohydrolase, C-terminal domain
beta-Lactamase/D-ala carboxypeptidase
beta-Phosphoglucomutase-like
Biotin repressor-like
BRCA2 helical domain
BRCA2 tower domain
Bromodomain
Calcium ATPase, transduction domain A
Calcium ATPase, transmembrane domain M

Calmodulin-like
Calpain large subunit, catalytic domain (domain II)
Calpain large subunit, middle domain (domain III)
cAMP-binding domain
CAPPD, an extracellular domain of amyloid beta A4 protein
Cell cycle transcription factor e2f-dp
Cell division protein ZapA-like
Cellulases catalytic domain
Cellulose-binding domain family III
Centromere-binding
Cfr10I/Bse634I
Chaperone J-domain
Chemotaxis phosphatase CheZ
Chemotaxis protein CheA P1 domain
CheW-like
Cholesterol oxidase
Choline/Carnitine O-acyltransferase
Circadian clock protein KaiA, C-terminal domain
Class I alpha-1;2-mannosidase, catalytic domain
Class I aminoacyl-tRNA synthetases (RS), catalytic domain
Class II aminoacyl-tRNA synthetase (aaRS)-like, catalytic domain
Class-II DAHP synthetase
Class II glutamine amidotransferases
Class III anaerobic ribonucleotide reductase NRDD subunit
clathrin assemblies
Clostridium neurotoxins, catalytic domain
Clostridium neurotoxins, "coiled-coil" domain
Clostridium neurotoxins, C-terminal domain
Clostridium neurotoxins, the second last domain
CMD-like
Cobalamin adenosyltransferase
Cobalamin (vitamin B12)-binding domain
CO dE-Hydrogenase flavoprotein C-terminal domain-like
CO dE-Hydrogenase flavoprotein N-terminal domain-like

CO dE–Hydrogenase ISP C–domain like
CO dE–Hydrogenase molybdoprotein N–domain–like
Coiled–coil domain of nucleotide exchange factor GrpE
Cold shock DNA–binding domain–like
Colicin E3 receptor domain
Computational models partly based on NMR data
Conserved hypothetical protein MTH1747
CorA soluble domain–like
Coronavirus NSP8–like
Coronavirus S2 glycoprotein
COX17–like
Creatinase/aminopeptidase
Creatinase/prolidase N–terminal domain
Crotonase–like
Cryptochrome/photolyase FAD–binding domain
Cryptochrome/photolyase, N–terminal domain
CSE2–like
C–terminal domain of alpha and beta subunits of F1 ATP synthase
C–terminal domain of class I lysyl–tRNA synthetase
C–terminal domain of DFF45/ICAD (DFF–C domain)
C–terminal domain of eukaryotic peptide chain release factor subunit 1, ERF1
C–terminal domain of Ku80
C–terminal fragment of elongation factor SelB
C–terminal fragment of thermolysin
C–terminal UvrC–binding domain of UvrB
CUE domain
Cullin homology domain
Cullin repeat
Cyclin
Cyclophilin (peptidylprolyl isomerase)
Cytochrome b562
Cytochrome b of cytochrome bc1 complex (Ubiquinol–cytochrome c reductase)

Cytochrome c'–like
Cytochrome c oxidase subunit III–like
Cytochrome c oxidase subunit II–like, transmembrane region
Cytochrome c oxidase subunit I–like
Cytochrome P450
Cytochrome p450 reductase N–terminal domain–like
Cytoplasmic domain of inward rectifier potassium channel
D–aminoacid aminotransferase–like PLP–dependent enzymes
DBL homology domain (DH–domain)
Dcp2 box A domain
Decarboxylase
DE–Hydroquinate synthase, DHQS
delta–Endotoxin, C–terminal domain
delta–Endotoxin (insectocide), middle domain
delta–Endotoxin (insectocide), N–terminal domain
Designed four–helix bundle protein
Designed single chain threE–Helix bundle
DHN aldolase/epimerase
Dihydrodipicolinate reductase–like
Dimeric chorismate mutase
Dimerisation domain of CENP–B
Dimerization–anchoring domain of cAMP–dependent PK regulatory subunit
DinB–like
Diol dE–Hydratase, gamma subunit
DLC
DNA–binding N–terminal domain of transcription activators
DNA–binding protein Mj223
DNA damage–inducible protein DinI
DnaK suppressor protein DksA, alpha–hairpin domain
DNA ligase/mRNA capping enzyme postcatalytic domain
DNA polymerase I
DNA polymerase III clamp loader subunits, C–terminal

domain
DnaQ-like 3'-5' exonuclease
DNA repair protein MutS, domain I
DNA repair protein MutS, domain II
DNA repair protein MutS, domain III
DNA topoisomerase IV, alpha subunit
Domain of poly(ADP-ribose) polymerase
Domain of the SRP/SRP receptor G-proteins
Double Clp-N motif
double-SIS domain
E2 regulatory, transactivation domain
EF2458-like
EF-hand modules in multidomain proteins
eIF-2-alpha, C-terminal domain
eIF2alpha middle domain-like
Electron transfer flavoprotein-ubiquinone oxidoreductase-like
Elongation factor Ts (EF-Ts), dimerisation domain
EntA-Im
Enzyme IIa from lactose specific PTS, IIa-lac
Enzyme I of the PEP:sugar phosphotransferase system HPr-binding (sub)domain
EpoxidE-Hydrolase
Epsilon subunit of F1F0-ATP synthase C-terminal domain
Epsilon subunit of F1F0-ATP synthase N-terminal domain
ERP29 C domain-like
ERP29 N domain-like
ESAT-6 like
E-set domains of sugar-utilizing enzymes
ETF-QO domain-like
Eukaryotic DNA topoisomerase I, catalytic core
Eukaryotic DNA topoisomerase I, dispensable insert domain
Eukaryotic DNA topoisomerase I, N-terminal DNA-binding fragment
Eukaryotic type KH-domain (KH-domain type I)

Exocyst complex component
Exodeoxyribonuclease V beta chain (RecC), C-terminal domain
EXOSC10 HRDC domain-like
Extended AAA-ATPase domain
F1F0 ATP synthase subunit A
F1F0 ATP synthase subunit C
FAD-linked oxidases, N-terminal domain
FAD-linked reductases, N-terminal domain
FAD/NAD-linked reductases, dimerisation (C-terminal) domain
FAD/NAD-linked reductases, N-terminal and central domains
FAT domain of focal adhesion kinase
Fe,Mn superoxide dismutase (SOD), C-terminal domain
Fe,Mn superoxide dismutase (SOD), N-terminal domain
FemXAB nonribosomal peptidyltransferases
Ferredoxin domains from multidomain proteins
Ferritin
Fertilization protein
FHV B2 protein-like
Fibrinogen coiled-coil and central regions
FIS-like
FKBP12-rapamycin-binding domain of FKBP-rapamycin-associated protein (FRAP)
FKBP immunophilin/proline isomerase
Flagellar export chaperone FliS
FMN-linked oxidoreductases
Formate dE-Hydrogenase N, cytochrome (gamma) subunit
Formin homology 2 domain (FH2 domain)
Fumarate reductase respiratory complex cytochrome b subunit, FrdC
Fungal zinc peptidase
GABA-aminotransferase-like
GAPDH-like

GAT domain
Gated mechanosensitive channel
G domain-linked domain
Glutamate-cysteine ligase
Glutamyl tRNA-reductase catalytic, N-terminal domain
Glutamyl tRNA-reductase dimerization domain
Glutathione peroxidase-like
Glutathione S-transferase (GST), C-terminal domain
Glutathione S-transferase (GST), N-terminal domain
GlyceraldE-Hyde-3-phosphate dE-Hydrogenase-like, N-terminal domain
Glycerol-3-phosphate (1)-acyltransferase
Glycerol-3-phosphate dE-Hydrogenase
(Glycosyl)asparaginase
Glycosyltransferase family 36 C-terminal domain
Glycosyltransferase family 36 N-terminal domain
GMC oxidoreductases
G proteins
GreA transcript cleavage factor, C-terminal domain
GreA transcript cleavage protein, N-terminal domain
GRIP domain
GroEL chaperone, ATPase domain
GroEL-like chaperone, apical domain
GroEL-like chaperone, intermediate domain
Group Ⅱ chaperonin (CCT, TRIC), apical domain
Group Ⅱ chaperonin (CCT, TRIC), ATPase domain
Group Ⅱ chaperonin (CCT, TRIC), intermediate domain
Group I mobile intron endonuclease
Group V grass pollen allergen
GTP cyclohydrolase I
half-ferritin
Haloperoxidase (bromoperoxidase)
HAL/PAL-like
HCDH C-domain-like
Head domain of nucleotide exchange factor GrpE

Heat-inducible transcription repressor HrcA, N-terminal domain
Heat shock protein 70kD (HSP70), C-terminal subdomain
Heat shock protein 70kD (HSP70), peptide-binding domain
Hect, E3 ligase catalytic domain
Helical scaffold and wing domains of SecA
Hemerythrin
Hepatitis B viral capsid (hbcag)
Hermes dimerisation domain
Hermes transposase-like
HisE-like (PRA-PH)
Histidine kinase
HlyD-like secretion proteins
HMA, heavy metal-associated domain
HMD dimerization domain-like
HMG-box
Homeodomain
Homodimeric domain of signal transducing histidine kinase
HP1531-like
HR1 repeat
HrcA C-terminal domain-like
HRDC domain from helicases
HSC20 (HSCB), C-terminal oligomerisation domain
Hsp90 co-chaperone CDC37
HSP90 C-terminal domain
Hut operon positive regulatory protein HutP
Hyaluronate lyase-like catalytic, N-terminal domain
Hyaluronate lyase-like, central domain
Hyaluronate lyase-like, C-terminal domain
Hyaluronidase N-terminal domain-like
Hyaluronidase post-catalytic domain-like
Hybrid and chimeric proteins
Hybrid cluster protein (prismane protein)
Hypothetical protein D-63
Hypothetical protein MTH393

Hypothetical protein MTH677
Hypothetical protein Ta1238
Hypothetical protein TM1457
Hypothetical protein VC0424
Hypothetical protein YfhH
Hypothetical protein YhaI
Hypothetical Protein YjhP
IF2B-like
I/LWEQ domain
IMD domain
Influenza hemagglutinin (stalk)
Inositol monophosphatase/fructose-1,6-bisphosphatase-like
Inositol polyphosphate 5-phosphatase (IPP5)
Insect pheromone/odorant-binding proteins
Insert subdomain of RNA polymerase alpha subunit
Interferon-induced guanylate-binding protein 1 (GBP1), C-terminal domain
Interferons/interleukin-10 (IL-10)
IpaD-like
Iron-containing alcohol dE-Hydrogenase
Isoprenyl diphosphate synthases
ISY1 N-terminal domain-like
KaiB-like
L27 domain
Lactate & malate dE-Hydrogenases, C-terminal domain
Lambda integrase-like, catalytic core
lambda integrase N-terminal domain
Large subunit
L-aspartase/fumarase
LcrE-like
LDH N-terminal domain-like
LemA-like
Lesion bypass DNA polymerase (Y-family), catalytic domain
Lesion bypass DNA polymerase (Y-family), little finger

domain
LeuD-like
Lipoxigenase N-terminal domain
Long-chain cytokines
L-sulfolactate dE-Hydrogenase-like
Magnesium transport protein CorA, transmembrane region
Mago nashi protein
Malate synthase G
Malic enzyme N-domain
MarR-like transcriptional regulators
Mason-Pfizer monkey virus matrix protein
MazG-like
Mechanosensitive channel protein MscS (YggB), C-terminal domain
Mechanosensitive channel protein MscS (YggB), middle domain
Mechanosensitive channel protein MscS (YggB), transmembrane region
MED7 hinge region
Medium chain acyl-CoA dE-Hydrogenase-like, C-terminal domain
Medium chain acyl-CoA dE-Hydrogenase, NM (N-terminal and middle) domains
Membrane ion ATPase
Meta-cation ATPase, catalytic domain P
Metal cation-transporting ATPase, ATP-binding domain N
Methane monooxygenasE-Hydrolase, gamma subunit
Methicillin resistance protein FemA probable tRNA-binding arm
Methionine synthase domain
Methionine synthase SAM-binding domain
Methionyl-tRNA synthetase (MetRS), Zn-domain
Methyl-accepting chemotaxis protein (MCP) signaling domain
Methyl-coenzyme M reductase alpha and beta chain

C-terminal domain
Methyl-coenzyme M reductase alpha and beta chain N-terminal domain
Methylmalonyl-CoA mutase, N-terminal (CoA-binding) domain
Middle domain of eukaryotic peptide chain release factor subunit 1, ERF1
MIF4G domain-like
MIF-related
Mismatch glycosylase
MIT domain
Mitochondrial ATP synthase coupling factor 6
Mitochondrial glycoprotein MAM33-like
Mob1/phocein
modified HD domain
Molybdenum cofactor-binding domain
Molybdopterin synthase subunit MoaE
monodomain cytochrome c
monomeric chorismate mutase
Motor proteins
MPP-like
mRNA decapping enzyme-like
MTH1187-like
Multidrug efflux transporter AcrB pore domain; PN1, PN2, PC1 and PC2 subdomains
Multidrug efflux transporter AcrB TolC docking domain; DN and DC subdomains
Multidrug efflux transporter AcrB transmembrane domain
MxiH-like
Myb/SANT domain
Myosin S1 fragment, N-terminal domain
N-acetyl transferase, NAT
N-acylglucosamine (NAG) epimerase
NAD-binding domain of HMG-CoA reductase
NadC C-terminal domain-like

NadC N-terminal domain-like
NAD+-dependent DNA ligase, domain 3
NADH oxidase/flavin reductase
NADPH-cytochrome p450 reductase FAD-binding domain-like
NADPH-cytochrome p450 reductase-like
NagZ-like
Neurotransmitter-gated ion-channel transmembrane pore
NF-kappa-B/REL/DORSAL transcription factors, C-terminal domain
Nickel-containing superoxide dismutase, NiSOD
Nickel-iron hydrogenase, large subunit
NifU C-terminal domain-like
NifU/IscU domain
Nitric oxide (NO) synthase oxygenase domain
Nitrogenase iron protein-like
NKL-like
Non-heme 11 kDa protein of cytochrome bc1 complex (Ubiquinol-cytochrome c reductase)
Nonstructural protein ns2, Nep, M1-binding domain
Nop domain
NSP3 homodimer
N-terminal domain of adenylylcyclase associated protein, CAP
N-terminal domain of alpha and beta subunits of F1 ATP synthase
N-terminal domain of cbl (N-cbl)
N-terminal domain of enzyme I of the PEP:sugar phosphotransferase system
N-terminal domain of eukaryotic peptide chain release factor subunit 1, ERF1
N-terminal domain of the circadian clock protein KaiA
N-terminal domain of the delta subunit of the F1F0-ATP synthase
N-terminal, RNA-binding domain of nonstructural protein

NS1
Nuclear receptor ligand-binding domain
Nucleotide and nucleoside kinases
NusA extra C-terminal domains
Occludin/ELL domain
Ohr/OsmC resistance proteins
Oligosaccharide phosphorylase
OmpH-like
Orbivirus capsid
Orbivirus core
Outer membrane efflux proteins (OEP)
Outer surface protein C (OspC)
Oxygen-evolving enhancer protein 3,
P40 nucleoprotein
PadR-like
Pantothenate synthetase (Pantoate-beta-alanine ligase, PanC)
PAPS sulfotransferase
PDEase
PE
Penicillin-binding protein 2x (pbp-2x), c-terminal domain
Penicillin binding protein dimerisation domain
Penta-EF-hand proteins
PEP carboxykinase C-terminal domain
PEP carboxykinase N-terminal domain
Pepsin-like
PepX catalytic domain-like
PepX C-terminal domain-like
Periplasmic domain of cytochrome c oxidase subunit II
PF1790-like
PfkB-like kinase
PFOR PP module
PFOR Pyr module
PG0816-like
Phase 1 flagellin

PHBH-like
Phenylalanyl-tRNA synthetase (PheRS)
PhoB-like
Phoshoinositide 3-kinase (PI3K), catalytic domain
Phoshoinositide 3-kinase (PI3K) helical domain
Phosphate binding protein-like
Phosphoenolpyruvate carboxylase
PhosphonoacetaldE-HydE-Hydrolase-like
Phosphorelay protein-like
Phosphorelay protein luxU
Phosphoserine phosphatase RsbU, N-terminal domain
Photosystem I reaction center subunit XI, PsaL
Photosystems
PhoU-like
PIN domain
Plant invertase/pectin methylesterase inhibitor
Plant lipoxigenases
PLC-like (P variant)
Pleckstrin-homology domain (PH domain)
Poly(ADP-ribose) polymerase, C-terminal domain
Poly A polymerase C-terminal region-like
Poly A polymerasE-Head domain-like
Polynucleotide phosphorylase/guanosine pentaphosphate synthase (PNPase/GPSI), domain 3
Polyphosphate kinase C-terminal domain
Potassium channel NAD-binding domain
POU-specific domain
PPE
PPK middle domain-like
PPK N-terminal domain-like
Predicted hydrolases Cof
Prefoldin
Pre-protein crosslinking domain of SecA
PriB N-terminal domain-like
Prion-like

Prokaryotic DksA/TraR C4-type zinc finger
Prokaryotic phospholipase A2
Prokaryotic type I DNA topoisomerase
Prokaryotic type KH domain (KH-domain type II)
Proteasome subunits
Proteinase/alpha-amylase inhibitors
Protein HNS-dependent expression A; HdeA
Protein kinases, catalytic subunit
Protein-L-isoaspartyl O-methyltransferase
Protein prenylyltransferase
Pseudo ankyrin repeat
PTS-regulatory domain, PRD
Putative anticodon-binding domain of alanyl-tRNA synthetase (AlaRS)
Putative thiamin/HMP-binding protein YkoF
Putative transcriptional regulator TM1602, C-terminal domain
PyrH-like
Pyrimidine 5'-nucleotidase (UMPH-1)
Pyruvate-ferredoxin oxidoreductase, PFOR, domain II
Pyruvate-ferredoxin oxidoreductase, PFOR, domain III
Pyruvate phosphate dikinase, central domain
Pyruvate phosphate dikinase, C-terminal domain
Pyruvate phosphate dikinase, N-terminal domain
R1 subunit of ribonucleotide reductase, C-terminal domain
R1 subunit of ribonucleotide reductase, N-terminal domain
Rabenosyn-5 Rab-binding domain-like
Rab geranylgeranyltransferase alpha-subunit, C-terminal domain
Rab geranylgeranyltransferase alpha-subunit, insert domain
Rad50 coiled-coil Zn hook
RAP domain
Ras-binding domain, RBD
RecA protein-like (ATPase-domain)
RecG, N-terminal domain

RecG "wedge" domain
Regulator of G-protein signaling, RGS
RelA/SpoT domain
Rel/Dorsal transcription factors, DNA-binding domain
Release factor
Reovirus components
Restriction endonuclease EcoO109IR
Retrovirus capsid protein C-terminal domain
Retrovirus capsid protein, N-terminal core domain
Ribonucleotide reductase-like
Ribosomal protein L19 (L19e)
Ribosomal protein L29 (L29p)
Ribosomal protein L7/12, C-terminal domain
Ribosomal protein L7/12, oligomerisation (N-terminal) domain
Ribosomal protein S15
Ribosomal protein S20
Ribosome-binding factor A, RbfA
Ribosome complexes
Ribosome recycling factor, RRF
RING finger domain, C3HC4
RNA polymerase
RNA polymerase alpha subunit dimerisation domain
RNA-polymerase beta
RNA-polymerase beta-prime
RNase III catalytic domain-like
Roadblock/LC7 domain
ROK
ROK associated domain
ROP protein
RuBisCo LSMT catalytic domain
RuBisCo LSMT C-terminal, substrate-binding domain
Rubredoxin
RWD domain
S100 proteins

Saposin B
SCF ubiquitin ligase complex WHB domain
ScpB/YpuH-like
Sec1/munc18-like (SM) proteins
Secreted chorismate mutase-like
Serine acetyltransferase
Serum albumin-like
Seryl-tRNA synthetase (SerRS)
SH2 domain
SH3-domain
Short-chain cytokines
Siah interacting protein N terminal domain-like
Sigma2 domain of RNA polymerase sigma factors
Sigma3 domain
Sigma4 domain
Signal peptide-binding domain
Single strand DNA-binding domain, SSB
SipA N-terminal domain-like
Siroheme synthase middle domains-like
Siroheme synthase N-terminal domain-like
Small-conductance potassium channel
Small subunit
Smc hinge domain
SopE-like GEF domain
Spectrin repeat
SPy1572-like
SRP alpha N-terminal domain-like
Stabilizer of iron transporter SufD
Staphylocoagulase
STAT
STAT DNA-binding domain
Substrate-binding domain of HMG-CoA reductase
Subtilases
Succinate dE-Hydrogenase/fumarate reductase flavoprotein, catalytic domain

Succinate dE-Hydrogenase/fumarate reductase flavoprotein C-terminal domain
Succinate dE-Hydrogenase/fumarate reductase flavoprotein N-terminal domain
Succinate dE-Hydrogenase/Fumarate reductase transmembrane subunits (SdhC/FrdC and SdhD/FrdD)
Superantigen MAM
Swaposin
Synatpobrevin N-terminal domain
T7 RNA polymerase
Tandem AAA-ATPase domain
TAZ domain
TBP-associated factors, TAFs
TENA/THI-4
Terpene synthases
Terpenoid cyclase C-terminal domain
Terpenoid cyclase N-terminal domain
Tetracyclin repressor-like, C-terminal domain
Tetracyclin repressor-like, N-terminal domain
Tetratricopeptide repeat (TPR)
Thermolysin-like
Tim10/DDP
TK-like PP module
TK-like Pyr module
Top domain of virus capsid protein
Topoisomerase V catalytic domain-like
Topoisomerase V repeat domain
TorD-like
Transcriptional repressor TraM
Transcription factor IIA (TFIIA), alpha-helical domain
Transcription factor IIA (TFIIA), beta-barrel domain
Transcription factor IIB (TFIIB), core domain
Transcription factor NusA, N-terminal domain
Transcription factor STAT-4 N-domain
Transducin (alpha subunit), insertion domain

Transglutaminase core
Transglutaminase N–terminal domain
Transglutaminase, two C–terminal domains
Transketolase C–terminal domain–like
Translationally controlled tumor protein TCTP (histamine–releasing factor)
Translin
Translocated intimin receptor (tir) intimin–binding domain
TransmembranE–Helical fragments
Transposase IS200–like
Trigger factor ribosome–binding domain
TrkA C–terminal domain–like
TrmB–like
Tryptophan synthase beta subunit–like PLP–dependent enzymes
t–snare proteins
TS–N domain
Tumor suppressor gene product Apc
TyeA–like
Type II restriction endonuclease catalytic domain
Type II restriction endonuclease effector domain
Typo IV secretion system protein TraC
TyrA dimerization domain–like
Tyrosine–dependent oxidoreductases
Ubiquitin activating enzymes (UBA)
U–box
Upper collar protein gp10 (connector protein)
Urocanase
Vacuolar ATP synthase subunit C
Vanabin–like
Vanillyl–alcohol oxidase–like
Variant surface glycoprotein (N–terminal domain)
VBS domain
Vertebrate phospholipase A2
Viral DNA–binding domain

Virus ectodomain
Voltage–gated potassium channels
VPR protein fragments
VPS23 C–terminal domain
VPS28 C–terminal domain–like
VPS28 N–terminal domain
VPS37 C–terminal domain–like
VPS9 domain
V–type ATP synthase subunit C
XseB–like
YciF–like
YfiT–like putative metal–dependent hydrolases
YfmB–like
YggX–like
YhfA–like
YihX–like
YlxM/p13–like
YppE–like
Zn metallo–beta–lactamase
14–3–3 protein
1–deoxy–D–xylulose–5–phosphate reductoisomerase, C–terminal domain
2Fe–2S ferredoxin domains from multidomain proteins
3–Methyladenine DNA glycosylase III (MagIII)
5–carboxymethyl–2–hydroxymuconate isomerase (CHMI)
5' nucleotidase–like
5' to 3' exonuclease catalytic domain
5' to 3' exonuclease, C–terminal subdomain
6–phosphogluconate dE–Hydrogenase–like, N–terminal domain
ABC transporter ATPase domain–like
Aconitase B, N–terminal domain
Aconitase iron–sulfur domain
Actin/HSP70
Acyl–CoA binding protein

acyl-CoA oxidase C-terminal domains
acyl-CoA oxidase N-terminal domains
Adapter protein APS, dimerisation domain
Adenylation domain of NAD+-dependent DNA ligase
Adenylyl and guanylyl cyclase catalytic domain
Adenylylcyclase toxin (the edema factor)
a domain/subunit of cytochrome bc1 complex (Ubiquinol-cytochrome c reductase)
Aerobic respiration control sensor protein, ArcB
AF0060-like
AF0941-like
AF1104-like
AgoG-like
Alcohol dE-Hydrogenase-like, C-terminal domain
Alcohol dE-Hydrogenase-like, N-terminal domain
AldE-Hyde ferredoxin oxidoreductase, C-terminal domains
AldE-Hyde ferredoxin oxidoreductase, N-terminal domain
ALDH-like
Allosteric chorismate mutase
alpha-Amylases, C-terminal beta-sheet domain
alpha-catenin/vinculin
alpha-D-glucuronidasE-Hyaluronidase catalytic domain
alpha-D-glucuronidase, N-terminal domain
Alpha-hemoglobin stabilizing protein AHSP
alpha-Subunit of urease
alpha-subunit of urease, catalytic domain
Amidase signature (AS) enzymes
A middle domain of Talin 1
Aminoacid dE-Hydrogenase-like, C-terminal domain
Aminoacid dE-Hydrogenases
Aminoglycoside phosphotransferases
AmyC C-terminal domain-like
AmyC N-terminal domain-like
Amylase, catalytic domain
Animal lipoxigenases

Anticodon-binding domain of a subclass of class I aminoacyl-tRNA synthetases
Antioxidant defense protein AhpD
Anti-sigma factor AsiA
Apolipophorin-III
Apolipoprotein
Aquaporin-like
Archaeal DNA-binding protein
Arfaptin, Rac-binding fragment
Arginyl-tRNA synthetase (ArgRS), N-terminal 'additional' domain
Aristolochene/pentalenene synthase
Armadillo repeat
Arp2/3 complex 16 kDa subunit ARPC5
Arylsulfatase
Aspartate/ornithine carbamoyltransferase
Aspartokinase allosteric domain-like
ATP synthase
ATP synthase (F1-ATPase), gamma subunit
a tRNA synthase domain
Atu0492-like
Avirulence protein AvrPto
B12-dependent (class II) ribonucleotide reductase
Bacterial dinuclear zinc exopeptidases
Bacterial exopeptidase dimerisation domain
Bacterial GAP domain
Bacterial glucoamylase C-terminal domain-like
Bacterial glucoamylase N-terminal domain-like
Bacterial photosystem II reaction centre, L and M subunits
Bacterial tryptophan 2,3-dioxygenase
Bacteriophage CII protein
Bacteriorhodopsin-like
BAG domain
Band 7/SPFH domain
BAR domain

Barrier-to-autointegration factor, BAF
BAS1536-like
BC ATP-binding domain-like
BC C-terminal domain-like
Bcl-2 inhibitors of programmed cell death
BC N-terminal domain-like
Beta-D-glucan exohydrolase, C-terminal domain
beta-Lactamase/D-ala carboxypeptidase
beta-Phosphoglucomutase-like
Biotin repressor-like
BRCA2 helical domain
BRCA2 tower domain
Bromodomain
Calcium ATPase, transduction domain A
Calcium ATPase, transmembrane domain M
Calmodulin-like
Calpain large subunit, catalytic domain (domain II)
Calpain large subunit, middle domain (domain III)
cAMP-binding domain
CAPPD, an extracellular domain of amyloid beta A4 protein
Cell cycle transcription factor e2f-dp
Cell division protein ZapA-like
Cellulases catalytic domain
Cellulose-binding domain family III
Centromere-binding
Cfr10I/Bse634I
Chaperone J-domain
Chemotaxis phosphatase CheZ
Chemotaxis protein CheA P1 domain
CheW-like
Cholesterol oxidase
Choline/Carnitine O-acyltransferase
Circadian clock protein KaiA, C-terminal domain
Class I alpha-1;2-mannosidase, catalytic domain
Class I aminoacyl-tRNA synthetases (RS), catalytic domain

Class II aminoacyl-tRNA synthetase (aaRS)-like, catalytic domain
Class-II DAHP synthetase
Class II glutamine amidotransferases
Class III anaerobic ribonucleotide reductase NRDD subunit
clathrin assemblies
Clostridium neurotoxins, catalytic domain
Clostridium neurotoxins, "coiled-coil" domain
Clostridium neurotoxins, C-terminal domain
Clostridium neurotoxins, the second last domain
CMD-like
Cobalamin adenosyltransferase
Cobalamin (vitamin B12)-binding domain
CO dE-Hydrogenase flavoprotein C-terminal domain-like
CO dE-Hydrogenase flavoprotein N-terminal domain-like
CO dE-Hydrogenase ISP C-domain like
CO dE-Hydrogenase molybdoprotein N-domain-like
Coiled-coil domain of nucleotide exchange factor GrpE
Cold shock DNA-binding domain-like
Colicin E3 receptor domain
Computational models partly based on NMR data
Conserved hypothetical protein MTH1747
CorA soluble domain-like
Coronavirus NSP8-like
Coronavirus S2 glycoprotein
COX17-like
Creatinase/aminopeptidase
Creatinase/prolidase N-terminal domain
Crotonase-like
Cryptochrome/photolyase FAD-binding domain
Cryptochrome/photolyase, N-terminal domain
CSE2-like
C-terminal domain of alpha and beta subunits of F1 ATP synthase
C-terminal domain of class I lysyl-tRNA synthetase

C–terminal domain of DFF45/ICAD (DFF–C domain)
C–terminal domain of eukaryotic peptide chain release factor subunit 1, ERF1
C–terminal domain of Ku80
C–terminal fragment of elongation factor SelB
C–terminal fragment of thermolysin
C–terminal UvrC–binding domain of UvrB
CUE domain
Cullin homology domain
Cullin repeat
Cyclin
Cyclophilin (peptidylprolyl isomerase)
Cytochrome b562
Cytochrome b of cytochrome bc1 complex (Ubiquinol–cytochrome c reductase)
Cytochrome c'–like
Cytochrome c oxidase subunit III–like
Cytochrome c oxidase subunit II–like, transmembrane region
Cytochrome c oxidase subunit I–like
Cytochrome P450
Cytochrome p450 reductase N–terminal domain–like
Cytoplasmic domain of inward rectifier potassium channel
D–aminoacid aminotransferase–like PLP–dependent enzymes
DBL homology domain (DH–domain)
Dcp2 box A domain
Decarboxylase
DE–Hydroquinate synthase, DHQS
delta–Endotoxin, C–terminal domain
delta–Endotoxin (insectocide), middle domain
delta–Endotoxin (insectocide), N–terminal domain
Designed four–helix bundle protein
Designed single chain threE–Helix bundle
DHN aldolase/epimerase

Dihydrodipicolinate reductase–like
Dimeric chorismate mutase
Dimerisation domain of CENP–B
Dimerization–anchoring domain of cAMP–dependent PK regulatory subunit
DinB–like
Diol dE–Hydratase, gamma subunit
DLC
DNA–binding N–terminal domain of transcription activators
DNA–binding protein Mj223
DNA damage–inducible protein DinI
DnaK suppressor protein DksA, alpha–hairpin domain
DNA ligase/mRNA capping enzyme postcatalytic domain
DNA polymerase I
DNA polymerase III clamp loader subunits, C–terminal domain
DnaQ–like 3'–5' exonuclease
DNA repair protein MutS, domain I
DNA repair protein MutS, domain II
DNA repair protein MutS, domain III
DNA topoisomerase IV, alpha subunit
Domain of poly(ADP–ribose) polymerase
Domain of the SRP/SRP receptor G–proteins
Double Clp–N motif
double–SIS domain
E2 regulatory, transactivation domain
EF2458–like
EF–hand modules in multidomain proteins
eIF–2–alpha, C–terminal domain
eIF2alpha middle domain–like
Electron transfer flavoprotein–ubiquinone oxidoreductase–like
Elongation factor Ts (EF–Ts), dimerisation domain
EntA–Im
Enzyme IIa from lactose specific PTS, IIa–lac

Enzyme I of the PEP:sugar phosphotransferase system HPr-binding (sub)domain
EpoxidE-Hydrolase
Epsilon subunit of F1F0-ATP synthase C-terminal domain
Epsilon subunit of F1F0-ATP synthase N-terminal domain
ERP29 C domain-like
ERP29 N domain-like
ESAT-6 like
E-set domains of sugar-utilizing enzymes
ETF-QO domain-like
Eukaryotic DNA topoisomerase I, catalytic core
Eukaryotic DNA topoisomerase I, dispensable insert domain
Eukaryotic DNA topoisomerase I, N-terminal DNA-binding fragment
Eukaryotic type KH-domain (KH-domain type I)
Exocyst complex component
Exodeoxyribonuclease V beta chain (RecC), C-terminal domain
EXOSC10 HRDC domain-like
Extended AAA-ATPase domain
F1F0 ATP synthase subunit A
F1F0 ATP synthase subunit C
FAD-linked oxidases, N-terminal domain
FAD-linked reductases, N-terminal domain
FAD/NAD-linked reductases, dimerisation (C-terminal) domain
FAD/NAD-linked reductases, N-terminal and central domains
FAT domain of focal adhesion kinase
Fe,Mn superoxide dismutase (SOD), C-terminal domain
Fe,Mn superoxide dismutase (SOD), N-terminal domain
FemXAB nonribosomal peptidyltransferases
Ferredoxin domains from multidomain proteins
Ferritin
Fertilization protein

FHV B2 protein-like
Fibrinogen coiled-coil and central regions
FIS-like
FKBP12-rapamycin-binding domain of FKBP-rapamycin-associated protein (FRAP)
FKBP immunophilin/proline isomerase
Flagellar export chaperone FliS
FMN-linked oxidoreductases
Formate dE-Hydrogenase N, cytochrome (gamma) subunit
Formin homology 2 domain (FH2 domain)
Fumarate reductase respiratory complex cytochrome b subunit, FrdC
Fungal zinc peptidase
GABA-aminotransferase-like
GAPDH-like
GAT domain
Gated mechanosensitive channel
G domain-linked domain
Glutamate-cysteine ligase
Glutamyl tRNA-reductase catalytic, N-terminal domain
Glutamyl tRNA-reductase dimerization domain
Glutathione peroxidase-like
Glutathione S-transferase (GST), C-terminal domain
Glutathione S-transferase (GST), N-terminal domain
GlyceraldE-Hyde-3-phosphate dE-Hydrogenase-like, N-terminal domain
Glycerol-3-phosphate (1)-acyltransferase
Glycerol-3-phosphate dE-Hydrogenase
(Glycosyl)asparaginase
Glycosyltransferase family 36 C-terminal domain
Glycosyltransferase family 36 N-terminal domain
GMC oxidoreductases
G proteins
GreA transcript cleavage factor, C-terminal domain
GreA transcript cleavage protein, N-terminal domain

GRIP domain
GroEL chaperone, ATPase domain
GroEL-like chaperone, apical domain
GroEL-like chaperone, intermediate domain
Group II chaperonin (CCT, TRIC), apical domain
Group II chaperonin (CCT, TRIC), ATPase domain
Group II chaperonin (CCT, TRIC), intermediate domain
Group I mobile intron endonuclease
Group V grass pollen allergen
GTP cyclohydrolase I
half-ferritin
Haloperoxidase (bromoperoxidase)
HAL/PAL-like
HCDH C-domain-like
Head domain of nucleotide exchange factor GrpE
Heat-inducible transcription repressor HrcA, N-terminal domain
Heat shock protein 70kD (HSP70), C-terminal subdomain
Heat shock protein 70kD (HSP70), peptide-binding domain
Hect, E3 ligase catalytic domain
Helical scaffold and wing domains of SecA
Hemerythrin
Hepatitis B viral capsid (hbcag)
Hermes dimerisation domain
Hermes transposase-like
HisE-like (PRA-PH)
Histidine kinase
HlyD-like secretion proteins
HMA, heavy metal-associated domain
HMD dimerization domain-like
HMG-box
Homeodomain
Homodimeric domain of signal transducing histidine kinase
HP1531-like
HR1 repeat

HrcA C-terminal domain-like
HRDC domain from helicases
HSC20 (HSCB), C-terminal oligomerisation domain
Hsp90 co-chaperone CDC37
HSP90 C-terminal domain
Hut operon positive regulatory protein HutP
Hyaluronate lyase-like catalytic, N-terminal domain
Hyaluronate lyase-like, central domain
Hyaluronate lyase-like, C-terminal domain
Hyaluronidase N-terminal domain-like
Hyaluronidase post-catalytic domain-like
Hybrid and chimeric proteins
Hybrid cluster protein (prismane protein)
Hypothetical protein D-63
Hypothetical protein MTH393
Hypothetical protein MTH677
Hypothetical protein Ta1238
Hypothetical protein TM1457
Hypothetical protein VC0424
Hypothetical protein YfhH
Hypothetical protein YhaI
Hypothetical Protein YjhP
IF2B-like
I/LWEQ domain
IMD domain
Influenza hemagglutinin (stalk)
Inositol monophosphatase/fructose-1,6-bisphosphatase-like
Inositol polyphosphate 5-phosphatase (IPP5)
Insect pheromone/odorant-binding proteins
Insert subdomain of RNA polymerase alpha subunit
Interferon-induced guanylate-binding protein 1 (GBP1), C-terminal domain
Interferons/interleukin-10 (IL-10)
IpaD-like

Iron-containing alcohol dE-Hydrogenase
Isoprenyl diphosphate synthases
ISY1 N-terminal domain-like
KaiB-like
L27 domain
Lactate & malate dE-Hydrogenases, C-terminal domain
Lambda integrase-like, catalytic core
lambda integrase N-terminal domain
Large subunit
L-aspartase/fumarase
LcrE-like
LDH N-terminal domain-like
LemA-like
Lesion bypass DNA polymerase (Y-family), catalytic domain
Lesion bypass DNA polymerase (Y-family), little finger domain
LeuD-like
Lipoxigenase N-terminal domain
Long-chain cytokines
L-sulfolactate dE-Hydrogenase-like
Magnesium transport protein CorA, transmembrane region
Mago nashi protein
Malate synthase G
Malic enzyme N-domain
MarR-like transcriptional regulators
Mason-Pfizer monkey virus matrix protein
MazG-like
Mechanosensitive channel protein MscS (YggB), C-terminal domain
Mechanosensitive channel protein MscS (YggB), middle domain
Mechanosensitive channel protein MscS (YggB), transmembrane region
MED7 hinge region
Medium chain acyl-CoA dE-Hydrogenase-like, C-terminal domain
Medium chain acyl-CoA dE-Hydrogenase, NM (N-terminal and middle) domains
Membrane ion ATPase
Meta-cation ATPase, catalytic domain P
Metal cation-transporting ATPase, ATP-binding domain N
Methane monooxygenasE-Hydrolase, gamma subunit
Methicillin resistance protein FemA probable tRNA-binding arm
Methionine synthase domain
Methionine synthase SAM-binding domain
Methionyl-tRNA synthetase (MetRS), Zn-domain
Methyl-accepting chemotaxis protein (MCP) signaling domain
Methyl-coenzyme M reductase alpha and beta chain C-terminal domain
Methyl-coenzyme M reductase alpha and beta chain N-terminal domain
Methylmalonyl-CoA mutase, N-terminal (CoA-binding) domain
Middle domain of eukaryotic peptide chain release factor subunit 1, ERF1
MIF4G domain-like
MIF-related
Mismatch glycosylase
MIT domain
Mitochondrial ATP synthase coupling factor 6
Mitochondrial glycoprotein MAM33-like
Mob1/phocein
modified HD domain
Molybdenum cofactor-binding domain
Molybdopterin synthase subunit MoaE
monodomain cytochrome c
monomeric chorismate mutase
Motor proteins

MPP-like
mRNA decapping enzyme-like
MTH1187-like
Multidrug efflux transporter AcrB pore domain; PN1, PN2, PC1 and PC2 subdomains
Multidrug efflux transporter AcrB TolC docking domain; DN and DC subdomains
Multidrug efflux transporter AcrB transmembrane domain
MxiH-like
Myb/SANT domain
Myosin S1 fragment, N-terminal domain
N-acetyl transferase, NAT
N-acylglucosamine (NAG) epimerase
NAD-binding domain of HMG-CoA reductase
NadC C-terminal domain-like
NadC N-terminal domain-like
NAD+-dependent DNA ligase, domain 3
NADH oxidase/flavin reductase
NADPH-cytochrome p450 reductase FAD-binding domain-like
NADPH-cytochrome p450 reductase-like
NagZ-like
Neurotransmitter-gated ion-channel transmembrane pore
NF-kappa-B/REL/DORSAL transcription factors, C-terminal domain
Nickel-containing superoxide dismutase, NiSOD
Nickel-iron hydrogenase, large subunit
NifU C-terminal domain-like
NifU/IscU domain
Nitric oxide (NO) synthase oxygenase domain
Nitrogenase iron protein-like
NKL-like
Non-heme 11 kDa protein of cytochrome bc1 complex (Ubiquinol-cytochrome c reductase)
Nonstructural protein ns2, Nep, M1-binding domain

Nop domain
NSP3 homodimer
N-terminal domain of adenylylcyclase associated protein, CAP
N-terminal domain of alpha and beta subunits of F1 ATP synthase
N-terminal domain of cbl (N-cbl)
N-terminal domain of enzyme I of the PEP:sugar phosphotransferase system
N-terminal domain of eukaryotic peptide chain release factor subunit 1, ERF1
N-terminal domain of the circadian clock protein KaiA
N-terminal domain of the delta subunit of the F1F0-ATP synthase
N-terminal, RNA-binding domain of nonstructural protein NS1
Nuclear receptor ligand-binding domain
Nucleotide and nucleoside kinases
NusA extra C-terminal domains
Occludin/ELL domain
Ohr/OsmC resistance proteins
Oligosaccharide phosphorylase
OmpH-like
Orbivirus capsid
Orbivirus core
Outer membrane efflux proteins (OEP)
Outer surface protein C (OspC)
Oxygen-evolving enhancer protein 3,
P40 nucleoprotein
PadR-like
Pantothenate synthetase (Pantoate-beta-alanine ligase, PanC)
PAPS sulfotransferase
PDEase
PE

Penicillin-binding protein 2x (pbp-2x), c-terminal domain
Penicillin binding protein dimerisation domain
Penta-EF-hand proteins
PEP carboxykinase C-terminal domain
PEP carboxykinase N-terminal domain
Pepsin-like
PepX catalytic domain-like
PepX C-terminal domain-like
Periplasmic domain of cytochrome c oxidase subunit II
PF1790-like
PfkB-like kinase
PFOR PP module
PFOR Pyr module
PG0816-like
Phase 1 flagellin
PHBH-like
Phenylalanyl-tRNA synthetase (PheRS)
PhoB-like
Phoshoinositide 3-kinase (PI3K), catalytic domain
Phoshoinositide 3-kinase (PI3K) helical domain
Phosphate binding protein-like
Phosphoenolpyruvate carboxylase
PhosphonoacetaldE-HydE-Hydrolase-like
Phosphorelay protein-like
Phosphorelay protein luxU
Phosphoserine phosphatase RsbU, N-terminal domain
Photosystem I reaction center subunit XI, PsaL
Photosystems
PhoU-like
PIN domain
Plant invertase/pectin methylesterase inhibitor
Plant lipoxigenases
PLC-like (P variant)
Pleckstrin-homology domain (PH domain)
Poly(ADP-ribose) polymerase, C-terminal domain

Poly A polymerase C-terminal region-like
Poly A polymerasE-Head domain-like
Polynucleotide phosphorylase/guanosine pentaphosphate synthase (PNPase/GPSI), domain 3
Polyphosphate kinase C-terminal domain
Potassium channel NAD-binding domain
POU-specific domain
PPE
PPK middle domain-like
PPK N-terminal domain-like
Predicted hydrolases Cof
Prefoldin
Pre-protein crosslinking domain of SecA
PriB N-terminal domain-like
Prion-like
Prokaryotic DksA/TraR C4-type zinc finger
Prokaryotic phospholipase A2
Prokaryotic type I DNA topoisomerase
Prokaryotic type KH domain (KH-domain type II)
Proteasome subunits
Proteinase/alpha-amylase inhibitors
Protein HNS-dependent expression A; HdeA
Protein kinases, catalytic subunit
Protein-L-isoaspartyl O-methyltransferase
Protein prenylyltransferase
Pseudo ankyrin repeat
PTS-regulatory domain, PRD
Putative anticodon-binding domain of alanyl-tRNA synthetase (AlaRS)
Putative thiamin/HMP-binding protein YkoF
Putative transcriptional regulator TM1602, C-terminal domain
PyrH-like
Pyrimidine 5'-nucleotidase (UMPH-1)
Pyruvate-ferredoxin oxidoreductase, PFOR, domain II

Pyruvate-ferredoxin oxidoreductase, PFOR, domain III
Pyruvate phosphate dikinase, central domain
Pyruvate phosphate dikinase, C-terminal domain
Pyruvate phosphate dikinase, N-terminal domain
R1 subunit of ribonucleotide reductase, C-terminal domain
R1 subunit of ribonucleotide reductase, N-terminal domain
Rabenosyn-5 Rab-binding domain-like
Rab geranylgeranyltransferase alpha-subunit, C-terminal domain
Rab geranylgeranyltransferase alpha-subunit, insert domain
Rad50 coiled-coil Zn hook
RAP domain
Ras-binding domain, RBD
RecA protein-like (ATPase-domain)
RecG, N-terminal domain
RecG "wedge" domain
Regulator of G-protein signaling, RGS
RelA/SpoT domain
Rel/Dorsal transcription factors, DNA-binding domain
Release factor
Reovirus components
Restriction endonuclease EcoO109IR
Retrovirus capsid protein C-terminal domain
Retrovirus capsid protein, N-terminal core domain
Ribonucleotide reductase-like
Ribosomal protein L19 (L19e)
Ribosomal protein L29 (L29p)
Ribosomal protein L7/12, C-terminal domain
Ribosomal protein L7/12, oligomerisation (N-terminal) domain
Ribosomal protein S15
Ribosomal protein S20
Ribosome-binding factor A, RbfA
Ribosome complexes
Ribosome recycling factor, RRF

RING finger domain, C3HC4
RNA polymerase
RNA polymerase alpha subunit dimerisation domain
RNA-polymerase beta
RNA-polymerase beta-prime
RNase III catalytic domain-like
Roadblock/LC7 domain
ROK
ROK associated domain
ROP protein
RuBisCo LSMT catalytic domain
RuBisCo LSMT C-terminal, substrate-binding domain
Rubredoxin
RWD domain
S100 proteins
Saposin B
SCF ubiquitin ligase complex WHB domain
ScpB/YpuH-like
Sec1/munc18-like (SM) proteins
Secreted chorismate mutase-like
Serine acetyltransferase
Serum albumin-like
Seryl-tRNA synthetase (SerRS)
SH2 domain
SH3-domain
Short-chain cytokines
Siah interacting protein N terminal domain-like
Sigma2 domain of RNA polymerase sigma factors
Sigma3 domain
Sigma4 domain
Signal peptide-binding domain
Single strand DNA-binding domain, SSB
SipA N-terminal domain-like
Siroheme synthase middle domains-like
Siroheme synthase N-terminal domain-like

Small-conductance potassium channel
Small subunit
Smc hinge domain
SopE-like GEF domain
Spectrin repeat
SPy1572-like
SRP alpha N-terminal domain-like
Stabilizer of iron transporter SufD
Staphylocoagulase
STAT
STAT DNA-binding domain
Substrate-binding domain of HMG-CoA reductase
Subtilases
Succinate dE-Hydrogenase/fumarate reductase flavoprotein, catalytic domain
Succinate dE-Hydrogenase/fumarate reductase flavoprotein C-terminal domain
Succinate dE-Hydrogenase/fumarate reductase flavoprotein N-terminal domain
Succinate dE-Hydrogenase/Fumarate reductase transmembrane subunits (SdhC/FrdC and SdhD/FrdD)
Superantigen MAM
Swaposin
Synatpobrevin N-terminal domain
T7 RNA polymerase
Tandem AAA-ATPase domain
TAZ domain
TBP-associated factors, TAFs
TENA/THI-4
Terpene synthases
Terpenoid cyclase C-terminal domain
Terpenoid cyclase N-terminal domain
Tetracyclin repressor-like, C-terminal domain
Tetracyclin repressor-like, N-terminal domain
Tetratricopeptide repeat (TPR)
Thermolysin-like
Tim10/DDP
TK-like PP module
TK-like Pyr module
Top domain of virus capsid protein
Topoisomerase V catalytic domain-like
Topoisomerase V repeat domain
TorD-like
Transcriptional repressor TraM
Transcription factor IIA (TFIIA), alpha-helical domain
Transcription factor IIA (TFIIA), beta-barrel domain
Transcription factor IIB (TFIIB), core domain
Transcription factor NusA, N-terminal domain
Transcription factor STAT-4 N-domain
Transducin (alpha subunit), insertion domain
Transglutaminase core
Transglutaminase N-terminal domain
Transglutaminase, two C-terminal domains
Transketolase C-terminal domain-like
Translationally controlled tumor protein TCTP (histamine-releasing factor)
Translin
Translocated intimin receptor (tir) intimin-binding domain
TransmembranE-Helical fragments
Transposase IS200-like
Trigger factor ribosome-binding domain
TrkA C-terminal domain-like
TrmB-like
Tryptophan synthase beta subunit-like PLP-dependent enzymes
t-snare proteins
TS-N domain
Tumor suppressor gene product Apc
TyeA-like
Type II restriction endonuclease catalytic domain

Type II restriction endonuclease effector domain
Typo IV secretion system protein TraC
TyrA dimerization domain-like
Tyrosine-dependent oxidoreductases
Ubiquitin activating enzymes (UBA)
U-box
Upper collar protein gp10 (connector protein)
Urocanase
Vacuolar ATP synthase subunit C
Vanabin-like
Vanillyl-alcohol oxidase-like
Variant surface glycoprotein (N-terminal domain)
VBS domain
Vertebrate phospholipase A2
Viral DNA-binding domain
Virus ectodomain
Voltage-gated potassium channels

VPR protein fragments
VPS23 C-terminal domain
VPS28 C-terminal domain-like
VPS28 N-terminal domain
VPS37 C-terminal domain-like
VPS9 domain
V-type ATP synthase subunit C
XseB-like
YciF-like
YfiT-like putative metal-dependent hydrolases
YfmB-like
YggX-like
YhfA-like
YihX-like
YlxM/p13-like
YppE-like
Zn metallo-beta-lactamase

Figure S1: Alignment of the sequences of 1FSL, and those of 1R8J, 1XL3, 2FM9, 2NP5, and 2P06, respectively, based on the 3D structure alignment produced by Dalilite. A segment with capital letters denotes that the 3D structure of this segment is similar to that of the aligned segment of the query protein. A residue with the mark "†" constitutes a common residue pattern.

```
1FSL vs. 1R8J   Z score = 4.4
                E                         F                    G                       H
                †  †    †                                  †   ††  †                 †  ††  †
1FSL  --KLTGHAEKLFALVRDSAGQLKAS-GTVVA--daalgsvHAQKaVTDPQFVVVKEALLKTIKAAVG--DKWSDeLSRAWEVAYDELAAAIKKA
1R8J  ayESQKLHQAMQTSYREIVLSYFSPnSNLNQsidnfvnmaFFAD-VPVTKVVEIHMELMDEFAKKLRveGRSED-ILLDYRLTLIDVIAHLCEMyrrsip
                †   †   †                                  †   ††  †             †   †   †

1FSL vs. 1XL3   Z score = 2.0
                E                    F                     G                    H
              †  †    †                                †   ††  †                †  ††  †
1FSL  kLTGHAEKLFALVRDSAGQlkasgtvvadaalgsvhaqkavTDPQFVVVKEALLKTIKAAVgdkWSDELSRAWEVAYDELAAAIKK-------------a
1XL3  nPLQPLRDTYRDAVMGYQG-------iyaiwsdlqkrfpngDIDSVILFLQKALSADLQSQ---QSGSGREKLGIVISDLQKLXEFsvsdqvxgfwqffs
           †   †   †                                †  ††   †                   †  ††  †

1FSL vs. 2FM9   Z score = 2.9
                E                    F                    G                    H
              †  †    †                                †   ††  †                †  ††  †
1FSL  KLTGHAEKLFALVRDSAGQlkasgtvvadaalgsvHAQKAVTDPQFVVVKEALLKTIKAAVGDK-wSDELSRAWEVAYDELAAAIK------------------------------
2FM9  FPALIKQASLDALFKCGKD--------aealkevfTNSNNVAGKKAIMEFAGLFRSALNATSDSpeAKTLLMKVGAEYTAQIIKDGlkeksafgpwlpetkkaeaklenlekqlld
                                                        †      †            †          †

1FSL  --------------------------------------------------------------------------------------ka
2FM9  iiknntggelsklstnlvmqevmpyiasciehnfgctldpltrsnlthlvdkaaakavealdmchqkhlemqtliplllrnvfaqipa

1FSL vs. 2NP5   Z score = 4.4
                      E                                                  F                        G                  H
               †  †                                     †                                †   ††  †                  †
1FSL  KLTGHAE------klfaLVRD----------------------------------sagqlkasgtvvadaalgSVHAqkaVTDPQFVVVKEALLKTIKAAVG---DKWSDELSRAWE
2NP5  KLLARLSeverggdparALFAamsqllpldearsreahvxaafavraatspslaeirrktlftirtglsavlIGIG--tPEAETRAALLLATVDGLALDAIgspALYPPEYLEHAL
                                                             †  †   †                  †   ††                       †

       ††  †
1FSL  VAYDELAAAIKKA--
2NP5  DIQIGMILQGADVvp
         †  †

1FSL vs. 2P06   Z score = 3.2
                   E                     F                     G                        H
              †  †    †                                   †   ††  †                †  ††  †
1FSL  kltghaeklfalvrdsaGQLK---asgtvvadaalgsvhaqkAVTDPQFVVVKEALLKTIKAAVGDKW--SDELSRAWEVAYDELAAAIKKA-
2P06  ---------MdyfrlaeKFLRemhakymkrvsrpgntprpwfDFSEERLLSRLFEEXDELREAVEKEDweNLRDELLDVANFCMYLWGKLSVk
                                               †   †                      †  ††      †
```

Figure S2: Multiple alignment of each protein and its homologues. The accession numbers of homologues are presented in the right side of each figure. The histogram at the bottom of each figure denotes the number of substitutions of each residue. A red bar means a helix.

(a) Multiple alignment of the sequences of 1FSL and its homologues.

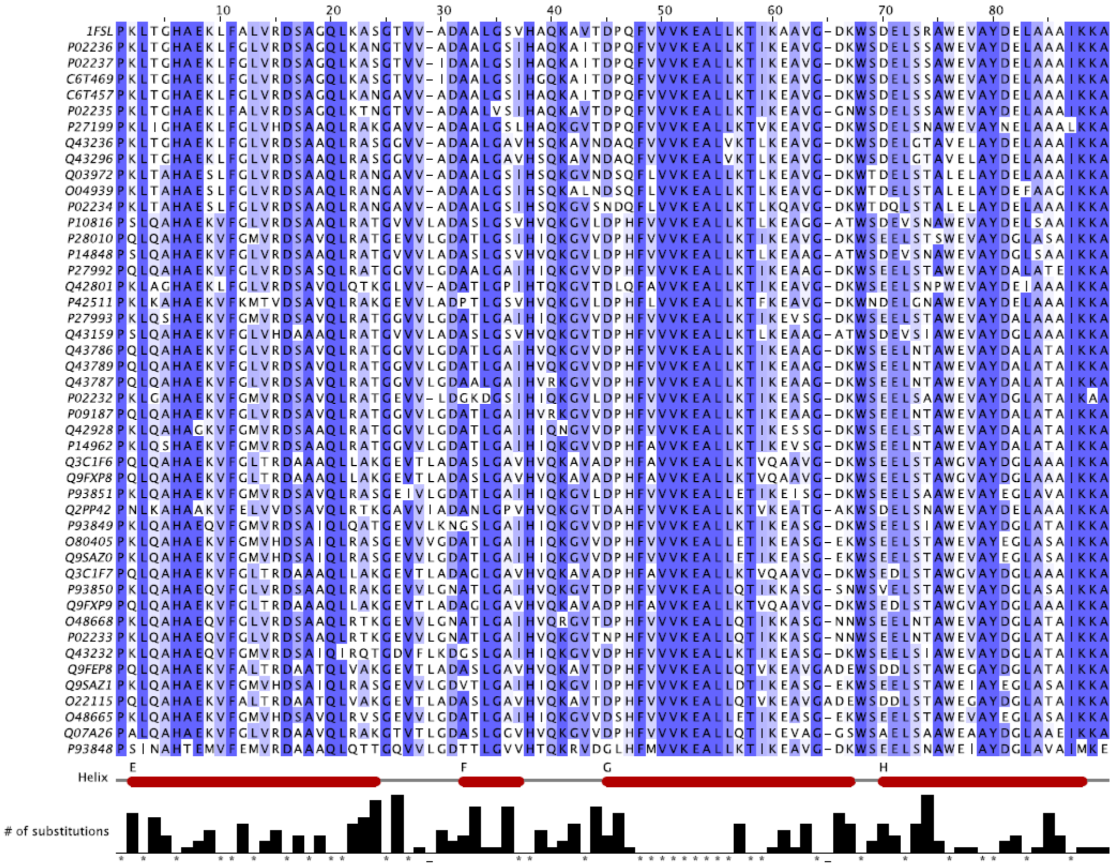

(b) Multiple alignment of the sequences of 1MBN and its homologues.

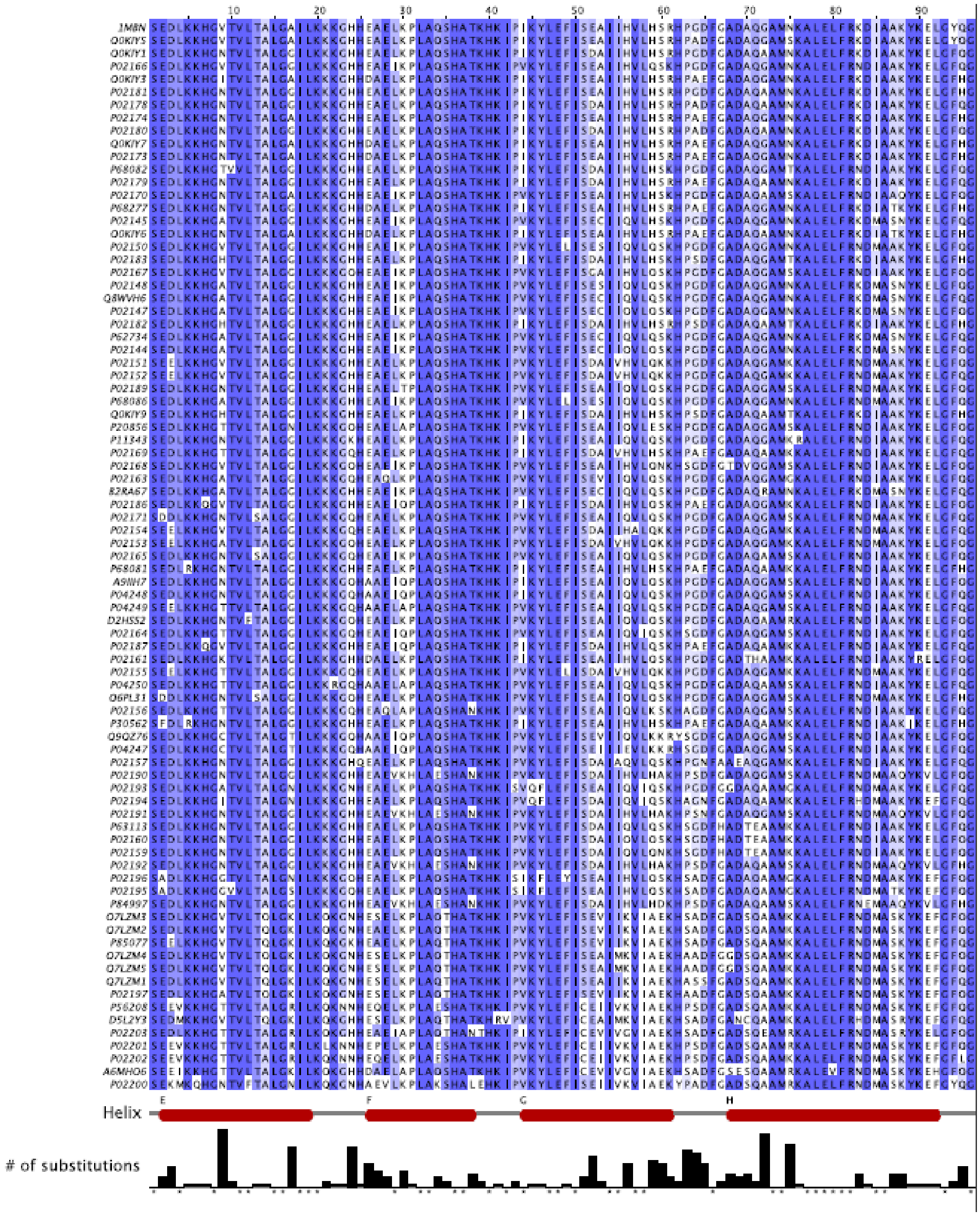

(c) Multiple alignment of the sequences of 1R8J and its homologues.

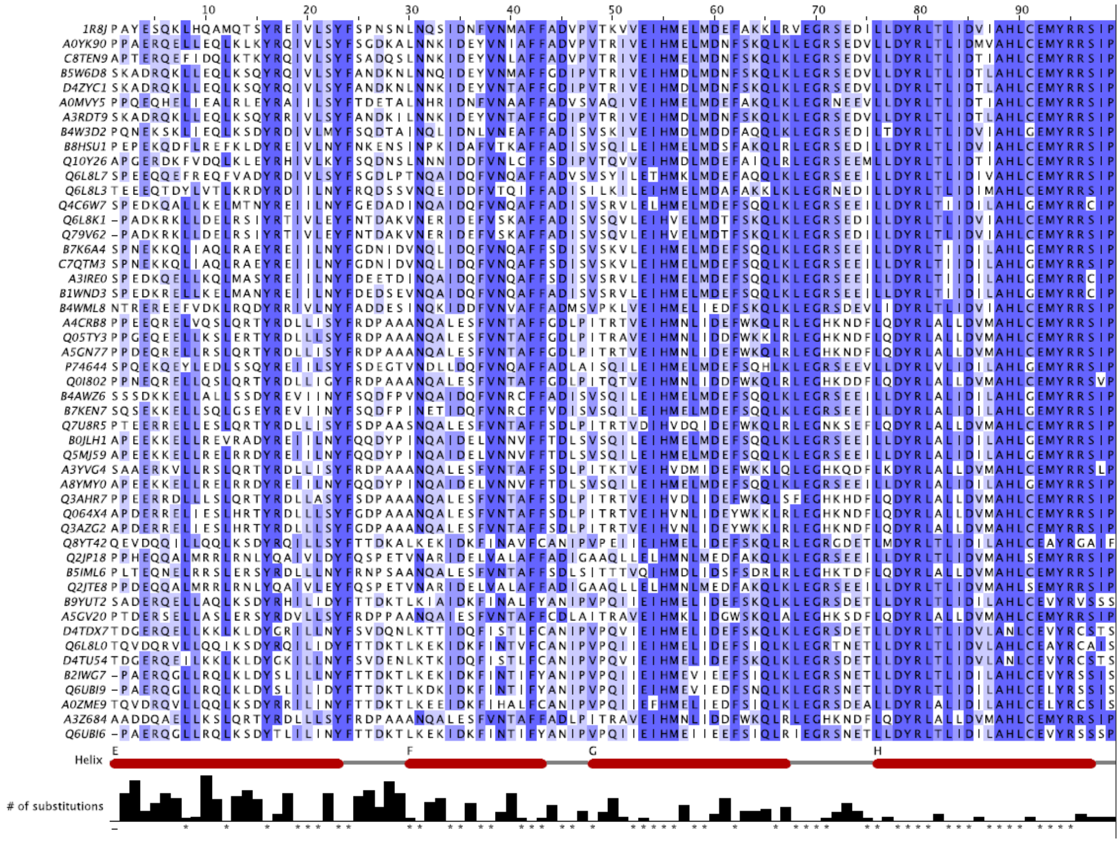

(d) Multiple alignment of the sequences of 1XL3 and its homologues.

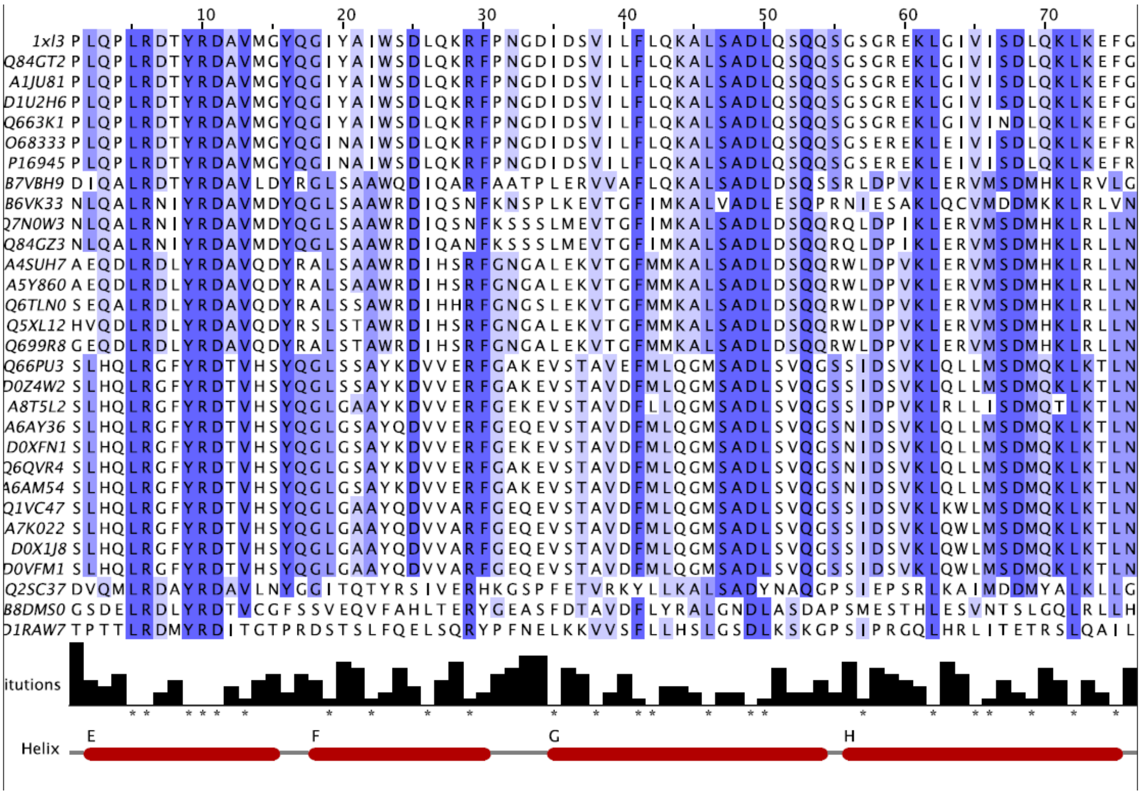

Supplement: Supplementary File 1 — Supplementary Information (PDF, 1525 KB) [file biomolecules-04-00268-s001.pdf]
